# Nature, Characteristics, and Reported Experiences of Medical Malpractice Disputes Among Egyptian Physicians Practicing in Egypt and Other Arab Countries: A Cross-Sectional Medicolegal Analysis of Psychological Distress, Somatic Symptoms, and Defensive Behavior

**DOI:** 10.3390/healthcare14213657

**Published:** 2026-10-28

**Authors:** Heba Abdel Samie Mohamed Hussein, Mohamed Hany, Ahmed ElShamarka, Mohamed H. Zidan, Hashem Altabbaa, Hagar Ahmad Aly Yassin, Ahmed Abokhozima, Mohammed Hassan Gaballah, Radwa Sehsah

**Affiliations:** 1 Forensic Medicine and Clinical Toxicology Department, Faculty of Medicine, Alexandria University, Alexandria 21561, Egypt; 2 Department of Surgery, Medical Research Institute, Alexandria University, Alexandria 21561, Egypt; mohamed.ashour@alexu.edu.eg; 3 Faculty of Medicine, Alexandria University, Alexandria 21561, Egypt; 4 The Research Papyrus Lab, Alexandria 21568, Egypt; 5 Al-Basheer Hospital, Amman 11151, Jordan; 6 ElMaamora Psychiatric Hospital, Egyptian Ministry of Health, Alexandria 21912, Egypt; 7 Jizan Forensic Medicine Center, Ministry of Health, Jazan 45142, Saudi Arabia; mgaballah@moh.gov.sa; 8 Industrial Medicine and Occupational Health, Public Health and Community Medicine Department, Faculty of Medicine, Mansoura University, Mansoura 35516, Egypt; radwa81@mans.edu.eg

**Keywords:** defensive medicine, Egypt, malpractice disputes, malpractice fear, medical liability system, psychological distress

## Abstract

**Background/Objectives:** Medical malpractice disputes may affect physicians’ well-being and clinical behavior. This study characterizes the medicolegal profile of malpractice disputes among Egyptian physicians practicing in Egypt and other Arab countries and examines how these disputes correlate with concurrent psychological, somatic, and defensive behavioral burdens. **Methods:** A cross-sectional online survey with nonprobability sampling was conducted from March to December 2025 among 586 practicing Egyptian physicians involved in at least one malpractice dispute. Participants completed the Kessler Psychological Distress Scale-6, the Impact of Event Scale-6, twelve items of the Defensive Medicine Behavior Scale, two items of the Malpractice Fear Scale, and a self-reported somatic symptom checklist. Multivariable regression identified independent correlates. **Results:** A lawsuit was the most serious dispute type for 51.4% of physicians, and management-related allegations were the most common (59.4%). Practice in Egypt rather than another Arab country was associated with procedure-related allegations (adjusted odds ratio 2.27; 95% confidence interval 1.49–3.45). Among resolved disputes, formal complaints (0.30; 0.18–0.49) and threats (0.45; 0.23–0.90) were less likely than lawsuits to end unfavorably for the physician. Recalling the dispute period, 62.3% had Kessler distress scores of 13 or more (mean 15.5 ± 6.4), with frequent post-traumatic stress symptoms (mean item score 2.7 ± 0.9) and somatic symptoms led by sleep disturbance (81.2%). High or very high defensive medicine scores were found in 92.0%. **Conclusions:** In this self-selected sample, malpractice disputes were associated with a heavy psychological, somatic, and behavioral burden. Medicolegal education, clearer complaint pathways, better documentation systems, and visible institutional peer support are policy considerations that need prospective evaluation.

## 1. Introduction

Medical malpractice refers to professional negligence by a physician, that is, a failure to meet the accepted standard of care of the profession that results in injury to the patient [1,2]. Medical malpractice disputes arise when clinical care is alleged to have deviated from accepted professional standards and to have resulted in patient harm. However, an allegation of malpractice is not equivalent to proven negligence. Adverse outcomes, unavoidable complications, medical errors, and legally actionable negligence are distinct medicolegal constructs; liability generally requires evidence of a breach in the standard of care, resulting harm, and a causal relationship between them [1,2]. This distinction is important because physicians may experience substantial professional and psychological consequences during the complaint or litigation process, even when negligence is not ultimately established.

In Egypt, the available malpractice literature remains fragmented. Local studies have mainly followed two lines of inquiry. The first consists of forensic or claims-based analyses describing malpractice allegations, specialty distribution, case characteristics, and legal or disciplinary outcomes [3,4,5,6,7,8,9]. The second consists mainly of cross-sectional physician surveys evaluating knowledge of medical liability, attitudes toward error disclosure, and defensive medicine practices. These studies have shown that malpractice concerns are clinically relevant in Egypt and that defensive medicine is common among physicians, particularly in high-pressure clinical settings. However, most prior work has focused either on the medicolegal profile of claims or on physicians’ knowledge and attitudes toward liability, rather than on the broader psychological, somatic, and behavioral consequences of being involved in a malpractice dispute [10,11,12,13,14,15,16].

International evidence suggests that complaints, claims, and malpractice litigation may be associated with anxiety, depressive symptoms, trauma-related reactions, somatic complaints, reduced professional confidence, and defensive clinical behaviors [17,18,19,20,21,22,23].

Defensive medicine is a change in clinical practice made mainly to reduce the physician’s own risk of complaints or litigation rather than to benefit the patient [18]. It takes two forms. Positive, or assurance, defensive medicine adds services of little clinical value, such as extra tests, imaging, consultations, referrals, or hospital admissions. Negative, or avoidance, defensive medicine means avoiding high-risk patients, complex cases, or high-risk procedures, or narrowing the scope of practice [18].

These consequences are closely related to the “second victim” phenomenon, which describes the emotional, cognitive, physical, and professional distress experienced by healthcare workers after adverse events, complaints, or perceived clinical failure. In this context, physicians may experience guilt, anxiety, intrusive recollections, sleep disturbance, loss of confidence, fear of blame, and changes in subsequent clinical behavior, even when negligence is not ultimately established [19,21,24,25].

Despite the relevance of this concept, Egyptian data remain limited regarding how malpractice disputes cluster with psychological distress, post-traumatic stress symptoms (PTSS), physical symptom burden, malpractice fear, and defensive practice within the same physician population.

Therefore, this study aimed to characterize the nature and reported experiences of medical malpractice disputes among Egyptian physicians, and to examine their associations with psychological distress, trauma-related symptoms, somatic symptoms, malpractice fear, and defensive medicine behaviors. By integrating medicolegal, psychological, somatic, and behavioral outcomes, this study seeks to provide a more comprehensive understanding of the burdens associated with malpractice-dispute exposure in the Egyptian healthcare context.

## 2. Materials and Methods

**Study design and setting:** This observational cross-sectional study was conducted from March to December 2025. It was an online survey with nonprobability sampling and is reported according to the STROBE (Strengthening the Reporting of Observational Studies in Epidemiology) checklist for cross-sectional studies (File S2).

**Study population:** The study targeted Egyptian physicians from various specialties who had been involved in at least one medical malpractice dispute and had worked in a healthcare facility in Egypt or in another Arab country for at least one year. Physicians who were on leave during the study period were excluded.

**Sample size:** The required sample size was estimated using the OpenEpi program, based on findings from a pilot study of 50 physicians (not included in the full-scale study), in which 68% reported high psychological distress. Assuming a 99% confidence level and 5% precision, the minimum required sample size was 577 physicians. A total of 588 questionnaires were submitted; after removal of two duplicate submissions, 586 physicians were included in the analysis.

**Study tool:** Data were collected using a predesigned, self-administered questionnaire developed on the basis of an extensive literature review and related studies (File S1). The questionnaire was in English, which is the language of undergraduate and postgraduate medical education in Egypt. Using English also allowed the Kessler Psychological Distress Scale-6 and the Impact of Event Scale-6 to be used in their original validated English versions without translation. The questionnaire included the following sections:

1. Sociodemographic characteristics, including age, sex (male or female, as reported by the participant), marital status, and highest educational qualification. The basic medical degree in Egypt is the Bachelor of Medicine and Bachelor of Surgery (MBBCh), which is required for a license to practice medicine. The highest qualification was recorded as the MBBCh only; a postgraduate diploma or master’s degree, both of which are clinical specialty degrees awarded by Egyptian universities after structured training and examinations; or a doctoral-level qualification, namely the Egyptian Doctor of Medicine (MD, a higher clinical doctorate obtained after the master’s degree), a Doctor of Philosophy (PhD), a fellowship, or board certification (for example, the Egyptian Fellowship or the Arab Board). Twelve participants (2.0%) were dentists; they were retained because their disputes follow the same complaint and litigation pathways.
2. Occupational characteristics, including specialty, current position, duration of employment, workplace, and country of current practice. Current positions follow the career grades used in Egyptian hospitals, and all three grades are licensed physicians. A resident is a physician in structured specialty training. A specialist has completed specialty training, usually with a postgraduate diploma or master’s degree, and practices independently within the specialty. A consultant holds the most senior clinical grade, usually after a doctoral degree, fellowship, or board certification, and carries full responsibility for patient care and for supervising junior staff. Comparable grades exist in the Arab countries where participants practiced. Country of practice was recorded as Egypt or another Arab country. The governorate and the urban or rural setting of practice were not recorded.
3. Characteristics of the medical malpractice dispute, including the number of disputes, the types of disputes faced, the nature of alleged negligence, the medical outcome, the legal outcome, and the support received during the dispute.

• Participants could select every type of dispute they had faced, and each participant was classified by the most serious type reported, in the order of lawsuit, formal complaint, and threat or blackmail. A threat or blackmail was an informal allegation of malpractice made directly against the physician by the patient or the patient’s relatives, who threatened to file a complaint or a lawsuit, or to expose the physician publicly, for example on social media, unless their demands, usually financial, were met. A formal complaint was a written complaint submitted by the patient or the family to the hospital administration, the health directorate or Ministry of Health, or the Medical Syndicate, and it was handled through an administrative or disciplinary investigation. A lawsuit was a legal claim filed with the public prosecution or a court, as a criminal case, a civil claim for compensation, or both. In this study, “malpractice dispute” is the general term that covers all three types.
• Medical negligence was defined as a breach of the physician’s duty of care. It is established when the care given falls below what a reasonably competent physician in the same specialty would provide, and the patient is harmed as a result [1,2]. The “nature of alleged negligence” describes what the complainant alleged and does not mean that negligence was proven. Some allegation types, such as breach of confidentiality, unethical conduct, and medical misadventure, are not negligence in the strict legal sense; they were included because they were raised as allegations within malpractice disputes.
• The nature of alleged negligence was classified into four distinct operational categories based on the phase of clinical delivery and the primary legal duty under evaluation:
  - Management-Related Allegations: Defined as failures in the overall execution, coordination, and oversight of the patient’s care pathway. This includes clinical management plans as well as professional-regulatory management (such as ethical conduct and confidentiality rules that govern the therapeutic relationship).
  - Diagnosis-Related Allegations: Defined as cognitive or investigative errors occurring during the clinical assessment and evaluative phase of care, including failure to communicate a diagnosis or finding to the patient.
  - Medication-Related Allegations: Restricted entirely to errors in pharmacological choice, administration, or side-effect monitoring.
  - Procedure-Related Allegations: Defined as events occurring directly during or as an immediate consequence of invasive or technical clinical interventions. This category encompasses technical and anesthetic execution, as well as essential legal-procedural prerequisites (informed consent issues), which were operationally grouped here because they were litigated concurrently with a physical procedure.
• Participants could select every allegation that applied to their disputes. Each allegation item was assigned to one of the four categories (Table S2), and a participant was counted in a category if at least one of its items was selected. The categories are therefore not mutually exclusive, and the number of physicians in a category can be smaller than the sum of its item counts. The authors developed this framework to group allegation items by the phase of care and the main legal duty in question.
• The medical outcome was the most severe harm reported: the patient’s death, the patient’s temporary or permanent disability, or emotional distress of the patient or family without death or disability. Participants could select every legal outcome that applied. The options separated court verdicts (innocence, a fine or compensation order, or a prison sentence) from administrative penalties, settlements with payment to the complainant, disputes dropped before reaching court, and disputes still in progress, and free-text answers were assigned to the closest option. For analysis, the legal outcome was grouped by its result for the physician. A favorable outcome included a court verdict of innocence, a dispute dropped before reaching court, or a dispute that ended without any action against the physician. An unfavorable outcome included a court conviction with a fine, compensation order, or prison sentence; an administrative penalty without a court decision; or a settlement or other payment by the physician. When more than one outcome was reported, an unfavorable outcome took precedence over a dispute still in progress, and a dispute still in progress took precedence over a favorable outcome. Favorable and unfavorable outcomes are therefore not equivalent to judicial acquittal and conviction. For threats, which are informal, the reported outcome reflects how the dispute ended for the physician, including any complaint or legal action that followed the threat. All dispute characteristics, including allegations and outcomes, were self-reported and were not verified against legal or institutional records.
• Support received was grouped into three categories:
  - Personal; Support from family and friends.
  - Workplace support: support from colleagues, supervisors, and hospital administration.
  - Professional support; support sought from a legal counselor/lawyer, forensic medicine expert, or the regional Medical Syndicate.

4. Psychological distress associated with malpractice disputes, assessed in relation to the participant’s most recent dispute using:
  ○ Kessler Psychological Distress Scale-6 (K6) [26]: a 6-item scale assessing psychological distress, including anxiety and depressive symptoms. The K6 is a widely used screening tool for nonspecific psychological distress whose cut-offs have been validated in general and clinical populations [26]. Each item is scored from 0 to 4, yielding a total score ranging from 0 to 24. Distress was categorized as low (0–7), moderate (8–12), and high (13–24). The cut-off of 13 or more is the validated threshold for serious psychological distress over the past 30 days [26]. Because participants rated the period of their dispute instead (see below), this cut-off and the lower bands were used for description only and do not estimate the prevalence of serious psychological distress. In the current study, Cronbach’s α was excellent (0.93).
  ○ Impact of Event Scale-6 (IES-6) [27]: a 6-item measure of PTSS covering intrusion, avoidance, and hyperarousal. It is a validated short form of the Impact of Event Scale-Revised [27]. Each item is scored from 0 to 4. The mean item score was calculated; higher scores indicate greater frequency and severity of PTSS. In the current study, Cronbach’s α was excellent (0.90).

The IES-6 was used as a brief screening measure of trauma-related symptoms and was not intended to establish a clinical diagnosis of post-traumatic stress disorder. Both the K6 and the IES-6 are self-reported screening tools. No participant was assessed by a psychiatrist or psychologist for this study, and no psychiatric diagnosis was made or taken from medical records. Participants answered both scales retrospectively, describing how they felt during the active period of their most recent dispute rather than during a fixed recent period, so the standard recall periods of the two scales (30 days for the K6 and 7 days for the IES-6) did not apply. The time since the dispute was not recorded. No cut-off was applied to the IES-6, and all regression models used the continuous scores of both scales.

5. Somatic symptom burden associated with malpractice disputes: Evaluated concurrently using a list of 15 self-reported somatic symptoms experienced during the index dispute, adapted from the established medicolegal framework of Arimany-Manso et al., 2018 [28]. Each symptom was scored dichotomously (yes = 1, no = 0), yielding a total cumulative somatic symptom burden score ranging from 0 to 15. Symptoms and medical conditions, including hypertension and other reported diagnoses, were self-reported and were not verified against medical records. The checklist has not been formally validated.
6. Practice and clinical behavioral modifications associated with malpractice disputes, assessed using:
  ○ Defensive Medicine Behavior Scale (DMBS) [29]: The original scale has 14 items on positive (assurance) and negative (avoidance) defensive behaviors, each scored on a 5-point Likert scale (1 to 5). It was developed in Turkey and has been used to measure defensive medicine among physicians of different surgical disciplines [29]. The questionnaire included 12 of the 14 items; the items on using imaging techniques more often and on placing more emphasis on informed consent forms were not included. The 12-item total score ranges from 12 to 60. For description only, it was divided into four equal bands: low (12–23), moderate (24–35), high (36–47), and very high (48–60). Cronbach’s α in the current study was 0.92 (0.87 for the seven positive items and 0.89 for the five negative items).
  ○ Malpractice fear: the questionnaire included two items of the Malpractice Fear Scale (MFS), which was developed and tested among emergency physicians [30]: “I am concerned that I will be involved in a malpractice case at any time” and “I feel pressured in my day-to-day practice by the threat of malpractice litigation.” Each item was scored on a 5-point Likert scale, and the malpractice fear score was their sum (range 2–10). This two-item score has not been validated as a short form of the MFS. Cronbach’s α in the current study was 0.90 (inter-item correlation 0.81). The DMBS and the malpractice fear score have no validated clinical cut-offs, and all regression models used the continuous scores.
7. Subjective assessment of the legal process of medical malpractice disputes.
8. Attitudes toward medical practice and career choice.

**Data collection and sampling strategy:** To target practicing clinicians across different healthcare sectors in Egypt and abroad, the structured questionnaire was created using Google Forms and distributed through closed, professional communication channels accessible exclusively to verified medical professionals. These included officially recognized governorate medical syndicate groups, institutional department networks across university and Ministry of Health hospitals, and verified postgraduate medical association forums. These groups covered several governorates, so recruitment was not limited to Alexandria. Physicians practicing in other Arab countries were included because about half of the physicians registered with the Egyptian Medical Syndicate work outside Egypt [31]; they share the same undergraduate training and professional registration as physicians practicing in Egypt. The sampling strategy was nonprobability, using mixed purposive and convenience sampling techniques. The invitation was addressed to practicing physicians, and the questionnaire opened only after two screening questions were answered “yes”: “I agree to participate in this research” and “I faced a medical malpractice claim at least once.” The word “claim” in the screening question covered all three dispute types. Responses were collected between 11 March and 5 December 2025; 178 (30.4%) were submitted by the end of September and 408 (69.6%) between October and December 2025. The invitation was shared in groups and forums whose membership changes over time, so the number of physicians who received it, and therefore the response rate, could not be determined. The form did not restrict submissions to one per person, and responses were anonymous. Three submissions with identical answers to every question, sent within 13 s of each other, were treated as one response, and the two duplicates were removed.

• **Statistical analysis:** Data were analyzed using IBM SPSS Statistics version 27. The analysis dataset was rebuilt from the original survey export, and the scale scores and principal models were re-derived independently with separately written code, with identical results. Specialty was not specified by two participants, who were excluded from analyses that included specialty; there were no other missing data. Continuous variables are reported as mean ± standard deviation (SD) or median and interquartile range (IQR), and categorical variables as frequencies and percentages. In the bivariate analysis, categorical variables were compared with the chi-squared test, or Fisher’s exact test when an expected count was below 5, and crude odds ratios (CORs) with 95% confidence intervals (CIs) were calculated. Continuous outcomes were compared with the Mann–Whitney U test for two groups and the Kruskal–Wallis test for three or more groups, and correlations were assessed with Spearman’s rho. To avoid excluding potential confounders too early, all variables with ***p*** < 0.20 in the bivariate analysis were entered together (Enter method) into the multivariable models, and every entered variable is shown in the tables with its reference category. Each allegation category was modeled with binary logistic regression (present = 1, absent = 0) among all 586 participants. The legal outcome was modeled as an unfavorable (1) versus a favorable (0) outcome among the 486 participants whose dispute had been resolved. As a sensitivity analysis, unfavorable and favorable outcomes were each modeled among all participants, with disputes still in progress counted as non-events. Psychological distress, PTSS, somatic symptom burden, defensive medicine behavior, and malpractice fear were modeled with multiple linear regression. Results are reported as adjusted odds ratios (AORs) or unstandardized coefficients (B) with 95% CIs. Two further sensitivity analyses repeated the defensive medicine model without the malpractice fear score and repeated every model with specialty in three groups (obstetrics and gynecology, other surgical specialties, and non-surgical specialties). Model diagnostics included the variance inflation factor (VIF) for collinearity, the Hosmer–Lemeshow test for calibration of the logistic models, Cook’s distance for influential observations, and the Breusch–Pagan test for heteroscedasticity in the linear models, with heteroscedasticity-robust (HC3) standard errors as a check. An association was considered robust when it remained significant after adjustment for specialty in three groups and, in the linear models, with HC3 standard errors; associations that did not meet this criterion are reported in the tables but were not interpreted further. A *p*-value < 0.05 was considered statistically significant.
• **Ethical considerations:** The study protocol was approved by the Research Ethics Committee of the Faculty of Medicine at Alexandria University (IRB number: 00012098, serial number: 0306388; date of approval: 12 November 2023). Informed consent was obtained electronically from all participants at the beginning of the questionnaire. Confidentiality and anonymity were maintained throughout the study. All methods were performed in accordance with the Declaration of Helsinki.

## 3. Results

### 3.1. Participant and Practice Characteristics

A total of 586 physicians participated, with a mean age of 43.9 ± 8.0 years; 50.2% were 43 years or older. Most were male (71.0%) and married (87.9%). Regarding the highest qualification, 2.4% held only the MBBCh, 55.3% held a postgraduate diploma or master’s degree, and 42.3% held an MD, PhD, a fellowship, or board certification. Most were from surgical specialties (70.8%), and two participants did not specify their specialty (Table S1). Obstetrics and gynecology was the largest specialty (32.9%), and 12 participants (2.0%) were dentists. Nearly half were consultants (49.3%), 46.6% were specialists, and 4.1% were residents. The mean employment duration was 17.6 ± 8.3 years, and 51.0% had worked for 17 years or more. Most physicians were practicing in Egypt (63.1%), whereas 36.9% were practicing in another Arab country. Governmental hospitals were the most reported workplace (53.1%), followed by private hospitals or clinics (49.8%) (Table 1).

### 3.2. Medicolegal Profile of Malpractice Disputes

The median number of malpractice disputes was 1. Most physicians reported involvement in only one dispute (61.3%), while 38.7% had experienced more than one. The most serious dispute type reported was a lawsuit for 51.4% of physicians, a formal complaint for 38.4%, and a threat or blackmail for 10.2%. The most frequently reported type of alleged negligence was management-related (59.4%), followed by procedure-related (35.2%), diagnosis-related (33.6%), and medication-related (7.7%) (Table S2).

Regarding medical consequences, 44.0% of disputes involved emotional distress in the patient or family, 31.6% involved disability, and 24.4% involved death. In terms of legal outcome, 61.6% of disputes ended favorably for the physician, most often because they were dropped before reaching court (38.7%), and 21.3% ended unfavorably, including court convictions (8.0%), administrative penalties (5.3%), and settlements or other payments (8.0%); 17.1% were still in progress. During the dispute, 76.1% of physicians reported receiving personal support, 69.6% received workplace support, and 42.3% sought professional support, whereas 1.9% reported receiving no support (Table 2).

**Table 2:** Number, nature, and outcome of medical malpractice disputes among participating physicians (*N* = 586).

Characteristic	n (%)
**Number of disputes, median (IQR)**	1 (1)
**Number of disputes**	
One	359 (61.3)
More than one	227 (38.7)
**Dispute type ^b^**	
Threat or blackmail	60 (10.2)
Formal complaint	225 (38.4)
Lawsuit	301 (51.4)
**Alleged negligence ^a^**	
Management-related	348 (59.4)
Procedure-related	206 (35.2)
Diagnosis-related	197 (33.6)
Medication-related	45 (7.7)
**Medical outcome**	
Patient’s death	143 (24.4)
Patient’s disability	185 (31.6)
Emotional distress of the patient or family	258 (44.0)
**Legal outcome ^c^**	
Favorable outcome	361 (61.6)
Court verdict of innocence	100 (17.1)
Dropped before court	227 (38.7)
Ended without action against the physician	34 (5.8)
Unfavorable outcome	125 (21.3)
Court conviction (fine, compensation, or prison)	47 (8.0)
Administrative penalty	31 (5.3)
Settlement or other payment by the physician	47 (8.0)
Still in progress	100 (17.1)
Court case	76 (13.0)
Investigation or other proceeding	24 (4.1)
**Support received ^a^**	
None	11 (1.9)
Personal	446 (76.1)
Workplace	408 (69.6)
Professional	248 (42.3)

IQR = interquartile range; n = number. All values were self-reported by participants. **^a^** Answers are not mutually exclusive ^b^ Most serious type of dispute the physician had faced (lawsuit, then formal complaint, then threat or blackmail). ^c^ Grouped by the result for the physician (Section 2). Favorable and unfavorable outcomes are not equivalent to judicial acquittal and conviction. When several outcomes were reported, an unfavorable outcome took precedence over a dispute still in progress, and a dispute in progress over a favorable outcome.

Physicians’ subjective appraisal of the legal process for dispute resolution was mixed. Nearly half of the participants considered the legal process fair (46.4%); however, 42.5% perceived it as weighted toward the plaintiff, and only 11.1% as weighted toward the defendant.

### 3.3. Independent Correlates of Malpractice Allegation Nature and Legal Outcome:

In the bivariate analyses (Tables S3 and S4), several sociodemographic and occupational characteristics demonstrated significant unadjusted associations with both the nature of the malpractice allegations and their final legal outcomes, achieving the screening threshold for multivariable inclusion.

After adjustment, physicians practicing in another Arab country had higher odds of diagnosis-related allegations (AOR 2.42; 95% CI 1.63–3.59) and lower odds of procedure-related allegations (AOR 0.44; 95% CI 0.29–0.67) than those practicing in Egypt. Non-surgical specialties were associated with diagnosis-related allegations (AOR 1.81; 95% CI 1.23–2.69), and female physicians with medication-related allegations (AOR 2.31; 95% CI 1.22–4.38). Working in a hospital affiliated with a specialized body was associated with lower odds of management-related (AOR 0.50) and diagnosis-related allegations (AOR 0.39) and higher odds of procedure-related allegations (AOR 2.33). Working in a governmental hospital showed a similar pattern for management-related (AOR 0.67) and procedure-related allegations (AOR 1.50), but these associations were weak and were attenuated after adjustment for specialty group (Table S12), so they are not interpreted further. Working in a university hospital was also associated with procedure-related allegations (AOR 1.66), whereas residents had lower odds of these allegations than consultants (AOR 0.27) (Table 3).

Among the 486 resolved disputes, formal complaints (AOR 0.30; 95% CI 0.18–0.49) and threats or blackmail (AOR 0.45; 95% CI 0.23–0.90) had lower odds of an unfavorable outcome than lawsuits (Table 4). Specialists had higher odds of an unfavorable outcome than consultants (AOR 1.80; 95% CI 1.05–3.09), but this association was not robust: it was not significant after adjustment for specialty group (*p* = 0.052; Table S12) or in the model of unfavorable outcomes that counted pending disputes as non-events (*p* = 0.060; Table S10). The associations for residents (AOR 2.52; 95% CI 0.75–8.50), more than one dispute (AOR 1.48; 95% CI 0.94–2.33), and procedure-related allegations (AOR 1.53; 95% CI 0.96–2.42) were not significant. In the sensitivity analysis that counted pending disputes as non-events (n = 584), formal complaints remained associated with lower odds of an unfavorable outcome, and formal complaints and threats or blackmail with higher odds of a favorable outcome. In that analysis only, more than one dispute was also associated with an unfavorable outcome, and specialist grade and procedure-related allegations with lower odds of a favorable outcome (Table S10).

### 3.4. Psychological Distress, Somatic Symptoms, and Defensive Medicine Behaviors Associated with Malpractice Disputes

Physicians involved in malpractice disputes exhibited a high concurrent burden of psychological, somatic, and professional distress (Table 5). Recalled psychological distress during the dispute was substantial, with a mean K6 score of 15.5; 62.3% of participants had scores of 13 or more, which describes distress recalled for the dispute period rather than the current prevalence of serious psychological distress. Self-reported PTSS were also frequent (mean item score = 2.7).

Self-reported somatic symptoms were highly prevalent among participants, most notably sleep disturbances (81.2%) and hypertension (21.0%), while 7.2% reported angina or myocardial infarction. Within the professional domain, involvement in disputes clustered with high levels of malpractice fear and defensive practice. The mean defensive medicine score was 46.1 of a possible 60, and the mean malpractice fear score was 8.1 of a possible 10. Notably, 92.0% of participants had high or very high defensive medicine scores, and 73.2% agreed that they were concerned about being involved in a malpractice case at any time (Table 5 and Table S5).

**Figure 1:**
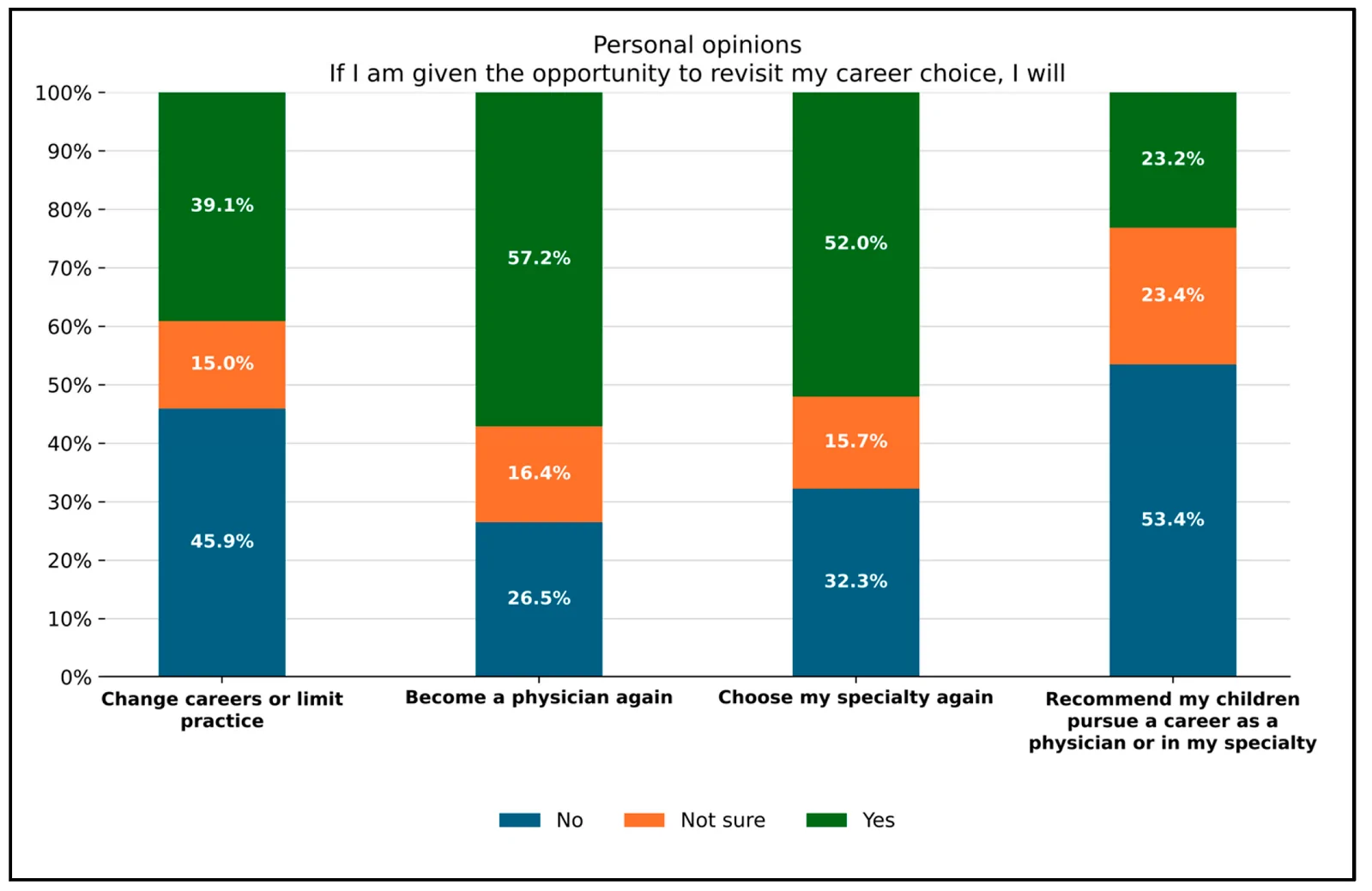
A stacked bar chart illustrating physicians’ personal opinions regarding career satisfaction, their willingness to re-enter the medical profession, and their likelihood of recommending the field to future generations.

Simultaneously, reported career-related attitudes suggested a substantial burden of professional dissatisfaction among physicians with a history of malpractice exposure. Although 57.2% and 52.0% of participants indicated they would choose to become physicians again or choose their specialty again, approximately 39% reported that, if given the opportunity, they would prefer to change careers or limit their practice scope. The most unfavorable pattern was observed regarding intergenerational recommendations: only 23.2% stated they would recommend that their children pursue a medical career or their own specialty, whereas 53.4% indicated they would not (Figure 1).

**Figure 2:**
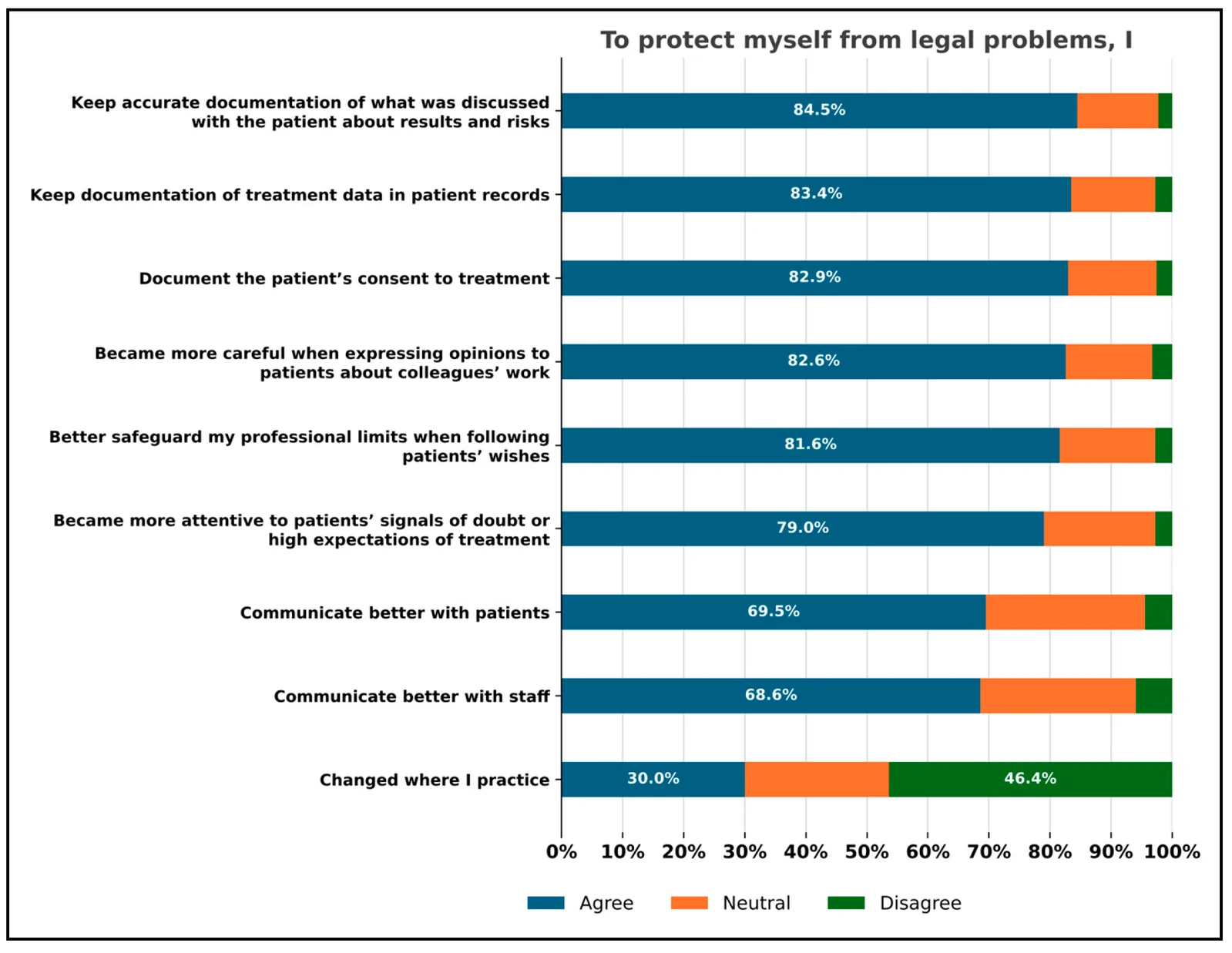
A horizontal bar chart displaying the frequency of self-reported changes in documentation, communication, and clinical practice adopted by physicians as protective measures against legal liability.

Participating physicians also frequently reported modifying their clinical practice in a self-protective manner. The most common changes concerned documentation: keeping accurate records of what was discussed with the patient about results and risks (84.5%), keeping treatment data in the patient record (83.4%), and documenting the patient’s consent (82.9%). Similar proportions reported greater caution when discussing colleagues’ work with patients (82.6%), better safeguarding of professional limits when following patients’ wishes (81.6%), and closer attention to patients’ signals of doubt or high expectations of treatment (79.0%). About two-thirds reported communicating better with patients (69.5%) and staff (68.6%). Changing the place of practice was less common: 30.0% reported such a change, and 46.4% disagreed that they had changed their place of practice (Figure 2).

### 3.5. Independent Correlates of Psychological and Somatic Distress:

Bivariate analyses were performed to examine the relationships between potential explanatory variables and the continuous outcomes: psychological distress, PTSS, somatic symptom burden, defensive medicine behavior, and malpractice fear. These preliminary analyses identified the variables that met the inclusion threshold (*p* < 0.20) for multivariable evaluation (Table S6–S9).

In the multivariable linear regression, female sex was associated with higher psychological distress (B 1.58; 95% CI 0.44–2.73), PTSS (B 0.20; 95% CI 0.04–0.37), and somatic symptom burden (B 1.15; 95% CI 0.63–1.66) (Table 6). Diagnosis-related allegations were associated with higher psychological distress and PTSS, and management-related allegations with higher PTSS. An age of 43 years or more and practice in a private hospital or clinic were associated with lower psychological distress, whereas specialists had higher PTSS than consultants. Legal outcome was not independently associated with psychological distress, PTSS, or somatic symptom burden. A higher somatic symptom burden was associated with being married, practicing in Egypt, and facing more than one dispute, and a lower burden with formal complaints, compared with lawsuits. Workplace support was also associated with a lower somatic symptom burden, but this association was not robust (*p* = 0.052 with robust standard errors and after adjustment for specialty group).

Notably, seeking professional support was associated with higher psychological distress (B 2.36; 95% CI 1.25–3.47) and PTSS (B 0.30; 95% CI 0.15–0.45), and personal support with slightly higher psychological distress (B 1.27; 95% CI 0.09–2.46) (Table 6).

This association should not be interpreted as evidence that professional support increased distress. Given the cross-sectional design, reverse causation and indication bias are plausible, as physicians experiencing greater distress or facing more complex medicolegal proceedings may have been more likely to seek professional support.

### 3.6. Independent Correlates of Perceived Malpractice Fear and Defensive Practice:

In the multivariable model for defensive medicine, the malpractice fear score had the strongest association with the DMBS score (B 3.32 per point; 95% CI 3.02–3.63), and PTSS showed a weaker positive association (B 0.93; 95% CI 0.02–1.84) that was not robust (*p* = 0.065 with robust standard errors) (Table 7). Physicians holding only the MBBCh also had higher scores, an estimate based on 14 participants. The two scores were strongly correlated (Spearman’s rho = 0.72; Table S9). When the malpractice fear score was removed from the model, higher psychological distress (B 0.23) and PTSS (B 2.51) were associated with higher DMBS scores, whereas residents, compared with consultants, and formal complaints, compared with lawsuits, were associated with lower scores (Table S11).

Higher malpractice fear scores were associated with facing more than one dispute (B 0.42), medication-related allegations (B 0.59), and disputes still in progress compared with favorable outcomes (B 0.60), and with higher psychological distress (B 0.04 per point) and PTSS (B 0.46) (Table 7).

### 3.7. Model Diagnostics

VIF values ranged from 1.01 to 1.63 in the logistic models and from 1.03 to 3.34 in the linear models, below the usual cut-off of 5. The Hosmer–Lemeshow test showed adequate calibration for all logistic models (*p* ≥ 0.16), and no observation had a Cook’s distance above 0.10. The Breusch–Pagan test indicated heteroscedasticity in the somatic symptom model (*p* = 0.029) and the malpractice fear model (*p* < 0.001). With robust standard errors, all associations in the malpractice fear model remained significant, whereas two borderline associations lost significance: workplace support with somatic symptom burden (*p* from 0.033 to 0.052) and PTSS with defensive medicine (*p* from 0.045 to 0.065). After adjustment for specialty in three groups (Table S12), governmental hospital practice was no longer associated with procedure-related allegations (*p* = 0.081) and became borderline for management-related allegations (*p* = 0.0498), specialist grade was no longer associated with an unfavorable legal outcome (*p* = 0.052), and workplace support remained non-significant for somatic symptom burden (*p* = 0.052). These associations are reported in the tables but are not interpreted further. All other associations remained significant, and obstetrics and gynecology was associated with higher odds of procedure-related allegations and of an unfavorable outcome, lower odds of diagnosis-related allegations, and a higher somatic symptom burden (Table S12).

## 4. Discussion

**Principal Findings**

Our findings demonstrate that medical malpractice disputes function concurrently as complex legal challenges and high-burden occupational experiences. A distinct pattern observed within our sample was the frequent reporting of repeated involvement in lawsuits and formal complaints, with management-related allegations emerging as the most prevalent category. While the reported legal outcomes of these disputes varied, with favorable outcomes, mostly disputes dropped before reaching court, occurring most frequently, our findings indicate that malpractice exposure is strongly associated with an elevated burden of psychological and somatic distress.

Because of the cross-sectional design, these findings should be interpreted strictly as associations. The study cannot determine whether malpractice disputes caused psychological distress, somatic symptoms, malpractice fear, or defensive medicine, nor can it determine whether physicians with higher baseline distress or defensive tendencies were more likely to perceive, recall, report, or become involved in medicolegal disputes. Accordingly, the observed clustering of distress, malpractice fear, somatic symptoms, and defensive practice should not be interpreted as a directional pathway, but rather as a concurrent pattern among physicians with prior malpractice-dispute exposure.

These cross-sectional data support the concept that navigating a malpractice dispute is closely intertwined with a clinician’s reported psychological well-being, somatic comfort, and perceived practice patterns. This relationship is methodologically vital because it suggests that participating physicians were not merely reflecting on unfavorable legal outcomes; rather, their reported distress scores cluster around the collective experience of accusation, peer review, and legal scrutiny. In other words, the core phenomenon captured within this cohort is not exclusively verified medical negligence, but rather a profound association with a complex liability process that co-occurs with significant professional and personal destabilization.

We believe this associative framing is especially vital within the Egyptian context. The local medicolegal environment cannot be reduced to a simplified civil compensation model alone; as the available Egyptian forensic literature demonstrates, medical disputes frequently involve overlapping professional, disciplinary, forensic, civil, and, at times, criminal pathways, as described by Rawash et al. [6]. This layered structural environment likely compounds the perceived threat of each complaint. Under these conditions, a participating physician may experience a dispute not merely as a localized challenge to clinical performance, but as a multi-dimensional threat to professional reputation, institutional standing, financial security, and, potentially, personal freedom. Our findings suggest that within this framework, experiencing a malpractice dispute is closely intertwined with a clinician’s daily working conditions, which may explain why histories of litigation strongly cluster with heightened fears, altered professional attitudes, and modified practice behaviors well beyond the immediate legal timeline [3,6,32].

Further implications arise from these data; since favorable outcomes were reported more often than unfavorable ones, court convictions accounted for only 8.0% of disputes, and unresolved cases remained common, combining allegations, clinical errors, and legally verified malpractice into a single analytical category should be avoided. Our study highlights the substantial perceived burden associated with entering a medicolegal framework in which a clinician’s past conduct is evaluated retrospectively, yet objective fault is not ultimately established. This distinction is far from a minor legal detail; it is key to understanding why the experience of a dispute itself strongly clusters with elevated distress and defensive behaviors, even when a final confirmation of clinical negligence is absent [3,10,33].

**Independent Correlates of Malpractice Allegation Types and Legal Outcomes**

Our results suggest that the distribution of allegation categories was not uniform across the cohort, but co-occurred significantly with specific physician and practice characteristics. While management-related allegations emerged as the most frequent category overall, diagnosis-related allegations clustered among physicians in non-surgical specialties, and medication-related allegations among female physicians. Additionally, practice within an Arab country outside Egypt was associated with higher odds of diagnosis-related allegations, whereas practice within Egypt and employment in university or specialized-body hospitals were associated with higher odds of procedure-related allegations. These patterns indicate that the observed distribution of allegation categories may be intertwined with structural variations in specialty-specific exposure, clinical workflows, institutional case mixes, and organizational contexts, rather than representing a uniform baseline of malpractice risk across all physicians [3,4,5,6,7,9].

Reported legal outcomes varied mainly with the type of dispute. Among resolved disputes, formal complaints and threats or blackmail had lower odds of an unfavorable outcome than lawsuits, and both were associated with higher odds of a favorable outcome when pending disputes were included. This pattern suggests that differing dispute pathways do not carry the same evidentiary weight or objective medicolegal gravity within the local legal framework. The higher odds of an unfavorable outcome among specialists did not persist after adjustment for specialty group, and procedure-related allegations were associated with a less favorable outcome only in the sensitivity analysis, so neither finding is interpreted as an independent association. Whether procedural disputes are more readily evaluated retrospectively, because procedures leave a clearer technical and documentary trail in operative records, perioperative notes, and follow-up documentation, remains a question for studies with verified legal records [3,6,9,34]. Because unfavorable outcomes combined court convictions, administrative penalties, and settlements, these associations describe how disputes ended for physicians, not judicial findings of negligence. Taken together, these findings argue against treating medical malpractice disputes as a single homogeneous phenomenon; rather, both the specific category of the allegation and the formal structure of the dispute co-occur with distinctly different profiles of physician medicolegal vulnerability.

**Co-Occurring Psychological Distress and Somatic Symptoms Associated with Medicolegal Exposure**

Our findings indicate that the burdens associated with malpractice disputes extend well beyond the legal domain to encompass a profound clustering of psychological distress and somatic manifestations. High recalled psychological distress scores and trauma-related symptoms were common within this cohort, and somatic symptoms, such as sleep disturbances, appetite changes, fatigue, and headaches, were also frequently reported. Because both scales were answered for the dispute period rather than for their standard recall periods, these scores describe recalled distress during the dispute and should not be read as the current prevalence of serious psychological distress or PTSS. These findings support the view that medicolegal exposure may be experienced by physicians as a prolonged occupational stressor rather than a discrete, isolated administrative event. The international literature similarly demonstrates that navigating complaints, litigation, and malpractice investigations strongly clusters with elevated anxiety, depressive symptoms, intrusive thoughts, hypervigilance, reduced professional confidence, and stress-related somatic symptoms among practicing clinicians [17,19,20,21,23].

These findings should not be interpreted as evidence of clinically diagnosed post-traumatic stress disorder, because the IES-6 is a brief screening instrument and not a diagnostic psychiatric interview, and it was answered retrospectively, outside its standard 7-day recall period.

Importantly, the pattern observed in our study was not limited to emotional discomfort alone. The concurrent reporting of psychological distress, PTSS, and an elevated somatic symptom burden highlights a broad, interconnected biopsychosocial pattern that co-occurs with a perceived medicolegal threat. Although we did not evaluate medical malpractice stress syndrome as a formal diagnostic construct, our findings align closely with prior descriptions of malpractice-related stress reactions and with the wider second-victim literature, which notes that physicians frequently report persistent emotional, cognitive, behavioral, and somatic responses following adverse clinical events or formal complaints [19,21,24,25]. This interpretation is consistent with our multivariable findings, in which diagnosis-related allegations were associated with higher psychological distress and PTSS, management-related allegations with higher PTSS, multiple disputes with a heavier somatic symptom burden, and higher psychological distress and PTSS with greater malpractice fear. These patterns indicate that this distress burden was not uniformly distributed across the sample, but instead clustered systematically alongside indicators of medicolegal severity and heightened perceptions of future vulnerability.

This issue remains insufficiently studied in Egypt. Most of the existing Egyptian literature has focused primarily on claim characteristics, physicians’ baseline knowledge of liability frameworks, or defensive medicine practices, rather than exploring the multidimensional psychological and somatic distress profiles experienced by clinicians navigating malpractice disputes [10,11,13]. In that context, our study adds needed empirical evidence from Egypt and may represent one of the few studies to examine medicolegal exposure through an integrated biopsychosocial lens. This is important because such burdens are unlikely to remain confined to physician well-being alone; when sustained over time, they may also influence confidence, communication, risk tolerance, and subsequent clinical behavior [21,22,23,25].

**Allegation Type and Legal Trajectory Shape Medicolegal Vulnerability**

In our study, management- and diagnosis-related allegations were associated with higher PTSS scores. One possible explanation is that such allegations may be experienced as direct challenges to clinical judgment, timeliness, and day-to-day professional competence, making them particularly destabilizing even when they are less technically discrete than procedure-based allegations. This interpretation should be made cautiously, however, because our cross-sectional data cannot determine why these allegation types were more strongly associated with trauma-related sequelae.

Legal trajectory also appeared to matter, mainly for malpractice fear. Compared with a favorable outcome, a dispute still in progress was associated with higher malpractice fear (Table 7), whereas an unfavorable outcome was not independently associated with psychological distress, PTSS, or somatic symptom burden (Table 6). This pattern is consistent with the broader literature showing that malpractice complaints and negligence processes may affect physicians not only through the final verdict, but also through the duration, uncertainty, and adversarial nature of the complaint process [17,20,21,22,23]. An unresolved case may prolong uncertainty and anticipatory stress. Thus, the medicolegal burden in this setting appears to be related not only to what was alleged, but also to how the dispute progresses and whether closure is achieved. In the Egyptian context, this may be especially relevant because malpractice disputes can pass through overlapping complaint, forensic, and disciplinary pathways before final resolution [3,6].

**Defensive Medicine as an Associated Practice Pattern in Malpractice Disputes**

Among the primary behavioral profiles identified in this study, defensive medicine stood out as one of the most prominent features. Within our cohort, defensive practice was highly prevalent, and defensive medicine scores correlated with psychological distress and PTSS (Spearman’s rho 0.35 for both). In the multivariable model, malpractice fear showed the strongest association with defensive practice, and psychological distress and PTSS were associated with defensive practice when malpractice fear was not in the model (Table S11). These findings support the interpretation that defensive medicine is not merely an incidental finding among physicians with medicolegal exposure, but one of the clearest behavioral patterns associated with it.

However, the strength of this association should be interpreted cautiously because malpractice fear and defensive medicine are conceptually related constructs (Spearman’s rho = 0.72). Both scales capture physician responses to anticipated complaint, blame, or litigation; therefore, the high magnitude of association may partly reflect construct overlap rather than a fully independent causal effect. Although collinearity diagnostics supported mathematical model stability, they do not eliminate conceptual overlap between related psychometric domains [18,35,36].

This interpretation is consistent with the international literature, which describes defensive medicine as a response to anticipated complaints, blame, or litigation rather than solely to clinical need [18,35,36,37]. That response may take the form of assurance behaviors, such as additional tests, referrals, or procedures, as well as avoidance behaviors, such as reluctance to manage high-risk patients or situations [18,35,36]. Studies of complaint processes have further shown that the psychological burden of investigations may spill over into routine clinical decision-making, thereby linking medicolegal stress to defensive practice [22,23,24]. In that sense, defensive medicine represents the point at which the burden of malpractice exposure moves from the legal arena into everyday patient care.

Our findings also align with Egyptian evidence. Previous studies from Egypt have shown that defensive medicine is common among physicians and is associated with malpractice claims, workplace violence, and specialty-specific pressures [10,11,16]. This local context is important because it suggests that defensive medicine in Egypt is not simply an individual coping style, but may represent a foreseeable adaptation to a medicolegal environment perceived as uncertain and insufficiently protective. In the bivariate analysis, physicians practicing in Egypt also had higher defensive medicine scores than those practicing in other Arab countries, although this difference did not persist after adjustment.

Not all reported behavioral modifications should be interpreted as exclusively maladaptive defensive medicine. Several commonly reported changes, including greater caution when discussing colleagues’ care, clearer professional boundaries, closer attention to patients’ doubts or expectations, and more effective communication with patients and staff, may also reflect adaptive professional development, reflective practice, and improved risk-aware care. However, because these changes were reported by physicians with prior malpractice-dispute exposure and co-occurred with high malpractice fear and defensive medicine scores, they may also contain a defensive component. A more balanced interpretation is therefore required: some behaviors may represent constructive responses that improve communication, documentation, expectation management, and shared decision-making, whereas others may reflect more problematic defensive practice, particularly when they involve unnecessary investigations, excessive referrals, avoidance of high-risk patients, or practice restriction. Future studies should distinguish between adaptive risk-management behaviors and maladaptive defensive medicine more explicitly [18,22,24,36]. The same applies to some positive items of the DMBS, such as spending more time with patients, explaining procedures in more detail, and keeping more detailed records. A high DMBS total score therefore combines beneficial risk-management behaviors with unnecessary use of services and avoidance, and it should not be read as entirely harmful.

**Documentation, Consent, Communication, and Disclosure Are Central to Defensibility**

A thorough malpractice investigation must focus on evidence. Our findings show that fears of malpractice extend beyond clinical errors to include concerns about the defensibility of medical actions under retrospective review. In medicolegal assessments, the medical record is vital, documenting care, available medical knowledge, decision rationale, patient interactions, and management of complications or adverse outcomes [32]. When medical records are incomplete, inconsistent, poorly timed, or inadequately standardized, care that could otherwise be justifiable becomes significantly more challenging to defend. Therefore, documentation should not be considered as a minor administrative task; rather, it constitutes a vital medicolegal protective measure [13,38].

This rationale extends to consent and disclosure practices as well. Analysis of claims in Egypt indicates that deficiencies in informed consent, falsification, and violations of patient privacy are not isolated incidents but rather consistent themes in malpractice investigations. Recent studies in Egypt suggest that while physicians may support the principles of disclosure and reporting, they often harbor concerns about the repercussions of such transparency, particularly in environments lacking robust institutional policies and tangible legal protections. This dynamic is critical for contextualizing our findings, as it clarifies why malpractice disputes continue to influence subsequent clinical interactions. When physicians believe that gaps in communication, inadequacies in consent, or incomplete records could be construed as concealment or negligence, their behavior tends to shift toward caution, defensiveness, and reduced confidence in patient interactions [3,13,14].

This concern is pronounced in the Egyptian setting. The extant literature highlights persistent issues, including limited standardization of reporting systems, the absence of mandatory liability insurance, restricted access to malpractice documentation, and underdeveloped mechanisms for institutional error review. These systemic deficiencies not only skew litigation metrics; they also shape physicians’ perceptions of the fairness and thoroughness of potential investigations into future complaints. Consequently, this contributes to the heightened vigilance observed in our data. In essence, what may be interpreted as anxiety or hypervigilance in clinical practice could be a rational adaptation to an environment in which defensibility is perceived as precarious [39].

Egyptian Law No. 13 of 2025 [40] provides important contextual background but should be interpreted cautiously in relation to the present findings. Because data collection took place from March to December 2025, overlapping with the publication of the law and the early phase of its implementation, participants’ reported experiences likely reflect the medicolegal climate that preceded full operationalization of these reforms. The law may therefore be better understood as a subsequent policy response to the types of systemic concerns highlighted by this study, including unclear liability pathways, limited institutional buffering, and fear of personally bearing the consequences of disputed outcomes [10,13,40]. Future studies should evaluate whether implementation of the new framework modifies physicians’ distress, malpractice fear, defensive practice, and perceived fairness of complaint resolution.

Differential vulnerability and cumulative burden were also evident.

Our results suggest that the multi-dimensional burdens associated with malpractice exposure were not uniformly distributed across the sample. Female physicians, younger practitioners, and those at earlier career stages demonstrated a heightened susceptibility to reporting elevated distress levels, while a history of repeated disputes and specific institutional contexts were concurrently relevant. These findings are best interpreted through a structural framework rather than through a lens of individual vulnerability. Junior physicians often operate with less institutional authority, may possess less familiarity with documentation standards and legal navigation, and typically have more restricted access to robust institutional advocacy when a formal complaint arises. Similarly, variations observed across physician sex may mirror unequal systemic scrutiny, established professional hierarchies, variable institutional support mechanisms, or skewed specialty distributions, rather than indicating any inherent individual vulnerability [9,22,23,41].

The cumulative aspect of the findings is also important. Repeated disputes were associated with a stronger pattern of malpractice fear and symptom burden rather than with clear adaptation. This argues against the assumption that physicians “get used” to complaints over time. A more plausible interpretation is that each new dispute may reactivate unresolved memories, reinforce vigilance, and deepen self-protective habits. Over time, the physician may become increasingly oriented toward avoiding future accusations, which in turn helps explain the persistent prominence of both fear and defensive behavior in the cohort. That cumulative pattern is one of the clearest reasons to view malpractice exposure as an occupational process rather than as an isolated event [25,41,42].

The observed association between private-sector practice and lower psychological distress warrants further interpretation. One plausible explanation is that private hospitals or clinics may offer physicians greater professional autonomy, more flexible scheduling, lower bureaucratic burden, and more direct control over patient selection, documentation processes, and follow-up pathways. In contrast, governmental and university hospitals often function within high-volume, resource-constrained environments, where physicians may face heavier patient loads, longer working hours, more complex emergency presentations, fragmented documentation systems, and greater administrative pressure [42]. University hospitals may additionally combine clinical service with teaching, supervision, research, and hierarchical responsibilities, which can intensify occupational strain when a malpractice dispute occurs [22,23,42].

However, this finding should be interpreted cautiously. Private practice is not inherently protective, and physicians in private settings may face distinct medicolegal vulnerabilities, including higher patient expectations, direct reputational consequences, financial pressures, and potentially greater exposure to complaints related to perceived service quality. Therefore, the lower distress observed among physicians working in private settings may reflect a combination of sector-level differences in workload, institutional support, case mix, documentation infrastructure, and perceived control over clinical practice rather than a direct protective effect of private employment itself [22,23,43,44]. These findings highlight the need for healthcare-sector-specific medicolegal support systems, particularly in high-volume governmental and university hospitals, where institutional buffering, peer support, legal guidance, and standardized documentation pathways may be especially important [10,22,44].

Although nearly half of the participants viewed the legal process as fair, a slight majority saw some imbalance, often favoring the plaintiff. This should not be over-interpreted as evidence of systemic bias, since the study assessed perception rather than actual adjudicative performance. However, perceived fairness remains highly relevant; a physician who expects partiality in the complaint process may experience increased anticipatory stress, decreased trust in institutional review, and a stronger tendency to modify practices defensively [10,17,24,37,45]. Accordingly, our data suggest that the malpractice burden may be related not only to outcome but also to how legitimate and balanced the process is experienced.

The findings regarding professional support require cautious interpretation. The positive association between seeking professional support and higher psychological distress or PTSS should not be interpreted as evidence that professional support worsened distress. A more plausible explanation is reverse causation or indication bias: physicians facing more severe distress, more complex disputes, or greater perceived legal threat may have been more likely to seek legal, forensic, syndicate, or other professional assistance. Moreover, the cross-sectional design does not allow assessment of whether support was timely, adequate, sustained, or effective. These findings therefore point less to harm from support itself and more to the need for structured, proactive, and accessible medicolegal and psychological support pathways before distress becomes severe [14,22,23,25].

**Career attitudes and professional disengagement after malpractice exposure**

Our results suggest that the multi-dimensional burdens associated with malpractice exposure are not isolated to elevated psychological distress and defensive practice patterns, but also strongly co-occur with distinct shifts within the domain of professional identity [17,20,21,22,23]. Although more than half of participants reported that they would choose medicine again and would select their specialty again, a substantial minority indicated that they would change careers or limit their practice if given the opportunity. Even more striking was the reluctance to recommend medicine or their own specialty to their children. Taken together, these findings suggest that the burdens associated with malpractice exposure reflect a broader profile of professional disillusionment rather than being limited to isolated or transient emotional distress [20,21,22,23]. This interpretation aligns closely with the broader literature, which demonstrates that navigating malpractice complaints and litigation strongly clusters with lower reported levels of physician well-being, diminished professional confidence, a compromised sense of security, and altered ongoing engagement with clinical work. The downstream burden of prolonged scrutiny, reputational threat, and repeated anticipation of future complaints may plausibly weaken physicians’ long-term attachment to practice. However, because of the cross-sectional design, malpractice exposure alone cannot be assumed to explain these career attitudes, as burnout, job strain, and dissatisfaction with working conditions may also contribute [42,43].

**Implications for Egyptian medicolegal reform and institutional practice**

The practical implications of these findings should be linked directly to the observed burden profile. Because psychological distress, malpractice fear, and defensive medicine clustered strongly within the cohort, interventions should not be limited to legal reform alone. At the institutional level, hospitals should develop clear pathways for early medicolegal guidance after complaints, structured peer support, access to legal or forensic advice, and standardized documentation and consent processes. These measures are particularly relevant for high-volume governmental and university hospitals, where workload, administrative complexity, and resource constraints may intensify distress during malpractice disputes. Because defensive medicine was highly prevalent, medicolegal education should also address risk communication, defensible documentation, disclosure practices, and rational investigation or referral decisions. These recommendations are policy considerations derived from the study’s cross-sectional associations between malpractice exposure, distress, professional support needs, malpractice fear, and defensive practice [3,7,10,40]. This study did not test them, and their effectiveness should be evaluated prospectively.

The recent Egyptian literature strongly supports this direction. Mostafa et al. [13] argue for reporting systems, insurance mechanisms, and educational efforts; Azab et al. [3] highlight the need for stronger monitoring, accreditation, and integrated claim investigation; and Egyptian defensive-medicine work underscores the importance of a comprehensive national medical liability framework. These recommendations align closely with the structure of our own findings. If fear of malpractice and defensive practice are sustained by ambiguity, weak institutional buffering, and inconsistent support, then coping advice alone will not be enough. Reform must also address the organizational and legal conditions that make each complaint feel personally threatening and professionally destabilizing [13,14,44].

## 5. Strengths and Limitations

This study has several strengths. It included a large sample of physicians who had personally faced a malpractice dispute, from many specialties and from practice settings in Egypt and other Arab countries. It examined medicolegal, psychological, somatic, and behavioral outcomes together, used scales with excellent internal consistency in this sample (Cronbach’s α 0.90–0.93), classified allegations with explicit operational definitions, reported every covariate of each multivariable model, and checked the models for collinearity, calibration, and influential observations, with sensitivity analyses for pending disputes, malpractice fear, and specialty. The analysis dataset and principal results were independently re-derived from the original survey export.

The study also has limitations. First, the cross-sectional design does not allow causal conclusions, and the direction of the observed associations cannot be determined. Unmeasured factors, such as workload, burnout, personality, and previous mental health, may also have confounded the associations. Many models were fitted, and the associations that did not persist with robust standard errors or specialty adjustment are reported but not interpreted; other isolated associations may also be chance findings. Second, sampling was nonprobability and online, through professional networks. Physicians who were more affected by their dispute may have been more likely to respond, which could overestimate distress and defensive practice. To our knowledge, no register of physicians involved in malpractice disputes exists in Egypt, so the size of the target population, the response rate, and the representativeness of the sample are unknown. The 586 participants represent less than 1% of the physicians registered with the Egyptian Medical Syndicate [31], and the results apply to physicians who chose to report a dispute, not to Egyptian physicians in general. The form did not limit submissions to one per person; exact duplicates were removed, but repeated participation with different answers cannot be excluded. The sample was also weighted toward high-risk specialties. Obstetrics and gynecology made up one third of participants, and surgical specialties made up 70.8%. Specialty group was tested as a correlate, and adjustment for specialty in three groups left most associations unchanged (Table S12), but analyses stratified by individual specialty were not possible because most specialties had few participants. Third, all data came from one self-administered questionnaire, so common-method bias may have inflated the associations between scales. Psychological distress and PTSS were measured with screening scales rather than clinical interviews, and no psychiatric diagnosis was made. Both scales were answered for the period of the most recent dispute rather than for their validated recall periods (the past 30 days for the K6 and the past 7 days for the IES-6), so the K6 cut-off was used descriptively; the proportion with scores of 13 or more is not a prevalence estimate, and the scores may be affected by recall. None of the scales had been validated specifically among Egyptian physicians or for online administration. Only 12 of the 14 DMBS items and 2 of the 6 MFS items were included in the questionnaire, so neither score is directly comparable with studies that used the full scales, and the two-item fear score has not been validated. Somatic symptoms and medical conditions were not verified against medical records, and the somatic checklist has not been formally validated. Recall bias is possible because participants reported on their most recent dispute, and the time since that dispute was not recorded. Allegations and legal outcomes were reported by participants and were not checked against court, syndicate, or hospital records; favorable and unfavorable outcomes also combined court verdicts with administrative decisions and settlements (Table 2). Fourth, the questionnaire was in English. This is the language of medical training in Egypt, but some participants may still have understood a few items differently from their intended meaning. Fifth, participants practicing in other Arab countries worked under legal and regulatory systems that differ from the Egyptian system. Country of practice was tested as a correlate in every analysis, but pooled results should be read with this difference in mind, and findings from physicians practicing abroad may not apply to those practicing in Egypt. Sixth, the governorate and the urban or rural setting of practice were not recorded, so regional differences in the frequency of malpractice disputes could not be examined. Future studies should record the place of practice and compare urban and rural settings. Finally, defensive medicine and malpractice fear are related constructs (Spearman’s rho = 0.72), and some DMBS items describe good practice, so the association between the two scores should be interpreted with care.

## 6. Conclusions

Our findings support the view that malpractice disputes are not transient legal inconveniences, but represent potentially traumatic occupational experiences that strongly cluster with how physicians report feeling, functioning, and practicing. A notable concurrent pattern within our data was the prominence of defensive medicine, which highlights the close interplay between profound emotional distress and modified clinical care patterns. Rather than viewing this issue solely as isolated individual suffering during a complaint, these findings emphasize the broader concern that unaddressed medicolegal stress co-occurs with systemic adjustments to medical practice. In the Egyptian setting, where clinicians frequently operate under high clinical demands, navigate fewer standardized medicolegal support structures, and utilize documentation systems that present distinct challenges for retrospective defense, these interconnected professional and personal burdens appear particularly pronounced. Because these findings come from a self-selected, cross-sectional sample, they do not establish that disputes caused these burdens, and the proposed institutional measures require testing in prospective studies. 

## Figures and Tables

**Table 1:** Sociodemographic and occupational characteristics of participating physicians (*N* = 586).

Characteristic	*n* (%)
**Age, years, mean ± SD**	43.9 ± 8.0
**Age group**	
<43 years	292 (49.8)
≥43 years	294 (50.2)
**Sex**	
Male	416 (71.0)
Female	170 (29.0)
**Marital status**	
Married	515 (87.9)
Not married	71 (12.1)
**Highest qualification**	
MBBCh only	14 (2.4)
Diploma or master’s degree	324 (55.3)
MD/PhD/fellowship/board	248 (42.3)
**Specialty**	
Surgical	415 (70.8)
Non-surgical	169 (28.8)
Not specified	2 (0.3)
**Current position**	
Resident	24 (4.1)
Specialist	273 (46.6)
Consultant	289 (49.3)
**Employment duration, years, mean ± SD**	17.6 ± 8.3
**Employment duration**	
≤10 years	114 (19.5)
11–15 years	150 (25.6)
16–20 years	159 (27.1)
>20 years	163 (27.8)
**Country of practice**	
Egypt	370 (63.1)
Another Arab country †	216 (36.9)
**Workplace ^a^**	
Governmental hospital	311 (53.1)
Private hospital or clinic	292 (49.8)
University hospital	108 (18.4)
Other hospital #	39 (6.7)

*N* = Number, SD = Standard Deviation, MBBCh = Bachelor of Medicine and Bachelor of Surgery, MD = Doctor of Medicine (Egyptian postgraduate clinical doctorate), PhD = Doctor of Philosophy. **^a^** Answers are not mutually exclusive. † Outside Egypt. # Hospitals affiliated with specialized bodies (Military Hospitals, General Authority for Health Insurance, Suez Canal Authority).

**Table 3:** Binary logistic regression analysis of sociodemographic and occupational correlates associated with the specific nature of alleged negligence in malpractice disputes among study participants (*N* = 586).

Correlate (Reference)	Management-Related			Procedure-Related			Diagnosis-Related			Medication-Related		
	B	*p*	AOR (95% CI)	B	*p*	AOR (95% CI)	B	*p*	AOR (95% CI)	B	*p*	AOR (95% CI)
Age ≥ 43 years (ref. <43 years)	–	–	–	−0.21	0.299	0.81 (0.55–1.20)	–	–	–	0.52	0.109	1.69 (0.89–3.20)
Female (ref. male)	–	–	–	–	–	–	0.33	0.107	1.39 (0.93–2.09)	**0.84**	**0.010**	**2.31 (1.22–4.38)**
Diploma or master’s degree (ref. MD/PhD/fellowship/board)	−0.17	0.338	0.84 (0.60–1.19)	–	–	–	–	–	–	–	–	–
MBBCh only (ref. MD/PhD/fellowship/board)	1.40	0.072	4.07 (0.88–18.79)	–	–	–	–	–	–	–	–	–
Non-surgical specialty (ref. surgical)	–	–	–	−0.14	0.508	0.87 (0.58–1.31)	**0.60**	**0.003**	**1.81 (1.23–2.69)**	0.55	0.090	1.74 (0.92–3.29)
Specialist (ref. consultant)	–	–	–	0.05	0.805	1.05 (0.69–1.60)	0.16	0.456	1.17 (0.78–1.76)	–	–	–
Resident (ref. consultant)	–	–	–	**−1.30**	**0.029**	**0.27 (0.09–0.87)**	0.77	0.091	2.17 (0.88–5.32)	–	–	–
Practice in another Arab country (ref. Egypt)	0.21	0.244	1.23 (0.87–1.75)	**−0.82**	**<0.001**	**0.44 (0.29–0.67)**	**0.88**	**<0.001**	**2.42 (1.63–3.59)**	–	–	–
Governmental hospital (ref. no)	**−0.41**	**0.020**	**0.67 (0.47–0.94)**	**0.40**	**0.038**	**1.50 (1.02–2.19)**	−0.28	0.152	0.76 (0.52–1.11)	−0.45	0.179	0.64 (0.33–1.23)
University hospital (ref. no)	–	–	–	**0.51**	**0.044**	**1.66 (1.01–2.73)**	−0.39	0.175	0.68 (0.38–1.19)	0.65	0.080	1.92 (0.92–4.00)
Other hospital # (ref. no)	**−0.70**	**0.039**	**0.50 (0.26–0.96)**	**0.85**	**0.017**	**2.33 (1.17–4.65)**	**−0.94**	**0.037**	**0.39 (0.16–0.95)**	–	–	–
**Constant**	0.64			−0.55			−1.18			−3.20		
**Model χ^2^ (df);** ***p***	18.1 (5); 0.003			45.3 (8); <0.001			62.8 (8); <0.001			18.0 (5); 0.003		
**Nagelkerke R^2^**	0.041			0.103			0.142			0.072		
**Correctly classified, %**	60.6			65.1			68.8			92.3		
**Hosmer–Lemeshow χ^2^ (8 df);** ***p***	7.80; 0.454			7.83; 0.451			10.29; 0.245			4.99; 0.759		
**VIF range**	1.01–1.06			1.01–1.39			1.02–1.27			1.01–1.09		
**Maximum Cook’s distance**	0.081			0.043			0.026			0.040		
**n (events)**	586 (348)			584 (206)			584 (195)			584 (45)		

AOR = adjusted odds ratio; B = unstandardized coefficient; CI = confidence interval; MBBCh = Bachelor of Medicine and Bachelor of Surgery; ref. = reference category; VIF = variance inflation factor. Each model includes all variables with *p* < 0.20 in the bivariate analysis (Table S3); – = not entered in that model. Bold: *p* < 0.05. Two participants without a specified specialty were excluded from models that included specialty (n = 584). # Hospitals affiliated with specialized bodies (Military Hospitals, General Authority for Health Insurance, Suez Canal Authority).

**Table 4:** Binary logistic regression analysis of correlates of an unfavorable versus a favorable legal outcome among resolved malpractice disputes (*N* = 486).

Correlate (Reference)	Unfavorable Versus Favorable Outcome		
	B	*p*	AOR (95% CI)
Diploma or master’s degree (ref. MD/PhD/fellowship/board)	0.08	0.791	1.08 (0.62–1.89)
MBBCh only (ref. MD/PhD/fellowship/board)	0.20	0.815	1.22 (0.23–6.40)
Non-surgical specialty (ref. surgical)	−0.46	0.080	0.63 (0.37–1.06)
Specialist (ref. consultant)	**0.59**	**0.032**	**1.80 (1.05–3.09)**
Resident (ref. consultant)	0.92	0.137	2.52 (0.75–8.50)
University hospital (ref. no)	−0.54	0.114	0.58 (0.30–1.14)
More than one dispute (ref. one)	0.39	0.088	1.48 (0.94–2.33)
Formal complaint (ref. lawsuit)	**−1.21**	**<0.001**	**0.30 (0.18–0.49)**
Threat or blackmail (ref. lawsuit)	**−0.79**	**0.023**	**0.45 (0.23–0.90)**
Procedure-related allegation (ref. no)	0.42	0.072	1.53 (0.96–2.42)
Constant	−1.08		
Model χ^2^ (df); *p*	50.2 (10); <0.001		
Nagelkerke R^2^	0.145		
Correctly classified, %	75.6		
Hosmer–Lemeshow χ^2^ (8 df); *p*	11.80; 0.160		
VIF range	1.05–1.63		
Maximum Cook’s distance	0.047		
n (events)	484 (124)		

AOR = adjusted odds ratio; B = unstandardized coefficient; CI = confidence interval; MBBCh = Bachelor of Medicine and Bachelor of Surgery; ref. = reference category; VIF = variance inflation factor. An unfavorable outcome (n = 125) was compared with a favorable outcome (n = 361); the 100 disputes still in progress were excluded (sensitivity analysis in Table S10), and two participants without a specified specialty were excluded from the model. The model includes all variables with *p* < 0.20 in the bivariate analysis (Table S4). Bold: *p* < 0.05.

**Table 5:** Psychological, Somatic, and Practice Burdens Associated with Medical Malpractice Disputes Among Participating Physicians (*N* = 586).

Measure	Value
**Psychological burden**	
**Kessler Psychological Distress Scale-6 (range 0–24)**	
Mean ± SD	15.5 ± 6.4
Median (IQR)	16.0 (10.0)
Low distress (0–7) ^d^, n (%)	79 (13.5)
Moderate distress (8–12) ^d^, n (%)	142 (24.2)
High distress (13–24) ^d^, n (%)	365 (62.3)
**Impact of Event Scale-6, mean item score (range 0–4)**	
Mean ± SD	2.7 ± 0.9
Median (IQR)	2.7 (1.3)
**Somatic burden**	
**Somatic symptoms (yes) ^a^, n (%)**	
Insomnia or poor-quality sleep	476 (81.2)
Changes in appetite or eating habits	458 (78.2)
Fatigue or reduced energy	436 (74.4)
Headache	370 (63.1)
Mental block	287 (49.0)
Changes in libido (particularly reduction)	234 (39.9)
Exacerbation of a previously diagnosed medical condition	210 (35.8)
Arterial hypertension	123 (21.0)
Diarrhea	78 (13.3)
Immunosuppression (recurrent infections)	72 (12.3)
Dermatological diseases	61 (10.4)
Angina or myocardial infarction	42 (7.2)
Bronchial asthma	25 (4.3)
Digestive hemorrhage	13 (2.2)
Stroke	9 (1.5)
Total somatic symptom score (range 0–15), median (IQR)	5 (4)
**Practice burden**	
**Defensive Medicine Behavior Scale, 12 items ^b^ (range 12–60)**	
Mean ± SD	46.1 ± 9.3
Median (IQR)	47.0 (14.0)
Low (12–23), n (%)	9 (1.5)
Moderate (24–35), n (%)	38 (6.5)
High (36–47), n (%)	248 (42.3)
Very high (48–60), n (%)	291 (49.7)
**Malpractice fear score, 2 items ^c^ (range 2–10)**	
Mean ± SD	8.1 ± 1.9
Median (IQR)	8.0 (3.0)
Agreed: concerned about being involved in a malpractice case, n (%)	429 (73.2)
Agreed: pressured by the threat of malpractice litigation, n (%)	428 (73.0)

IQR = interquartile range; n = number; SD = standard deviation. **^a^** Answers are not mutually exclusive. ^b^ The items on using imaging techniques more often and on placing more emphasis on informed consent forms were not part of the questionnaire. ^c^ Sum of two Malpractice Fear Scale items, each scored 1–5; agreed = agree or strongly agree. ^d^ Scores refer to the period of the most recent dispute, not to the past 30 days; the bands are descriptive and do not estimate the prevalence of serious psychological distress.

**Table 6:** Multivariable linear regression analysis of sociodemographic, occupational, and dispute-related correlates associated with psychological distress and somatic symptom burdens among study participants (*N* = 586).

Correlate (Reference)	Psychological Distress (K6)				PTSS (IES-6)				Somatic Symptom Score			
	B	SE	*p*	95% CI	B	SE	*p*	95% CI	B	SE	*p*	95% CI
Age ≥ 43 years (ref. <43 years)	**−1.68**	**0.78**	**0.031**	**−3.21, −0.16**	−0.12	0.11	0.249	−0.33, 0.09	–	–	–	–
Female (ref. male)	**1.58**	**0.58**	**0.007**	**0.44, 2.73**	**0.20**	**0.08**	**0.015**	**0.04, 0.37**	**1.15**	**0.26**	**<0.001**	**0.63, 1.66**
Not married (ref. married)	–	–	–	–	0.04	0.12	0.750	−0.19, 0.26	**−1.18**	**0.36**	**0.001**	**−1.89, −0.46**
Diploma or master’s degree (ref. MD/PhD/fellowship/board)	−0.41	0.64	0.529	−1.67, 0.86	−0.09	0.09	0.280	−0.27, 0.08	0.19	0.23	0.422	−0.27, 0.65
MBBCh only (ref. MD/PhD/fellowship/board)	1.69	2.00	0.397	−2.23, 5.62	0.17	0.28	0.543	−0.37, 0.71	0.20	0.76	0.787	−1.28, 1.69
Non-surgical specialty (ref. surgical)	–	–	–	–	–	–	–	–	−0.17	0.25	0.499	−0.66, 0.32
Specialist (ref. consultant)	1.01	0.72	0.163	−0.41, 2.43	**0.23**	**0.10**	**0.018**	**0.04, 0.43**	–	–	–	–
Resident (ref. consultant)	1.41	1.67	0.400	−1.88, 4.70	0.30	0.23	0.190	−0.15, 0.75	–	–	–	–
Employment 11–15 years (ref. ≤10 years)	0.77	0.80	0.338	−0.81, 2.34	−0.04	0.11	0.744	−0.25, 0.18	−0.20	0.34	0.550	−0.86, 0.46
Employment 16–20 years (ref. ≤10 years)	0.04	0.85	0.965	−1.63, 1.70	−0.09	0.12	0.462	−0.31, 0.14	−0.55	0.34	0.103	−1.22, 0.11
Employment > 20 years (ref. ≤10 years)	1.30	1.02	0.201	−0.69, 3.29	0.12	0.14	0.384	−0.15, 0.39	0.16	0.34	0.629	−0.50, 0.83
Practice in another Arab country (ref. Egypt)	−0.96	0.63	0.128	−2.20, 0.28	−0.16	0.09	0.063	−0.33, 0.01	**−0.85**	**0.26**	**0.001**	**−1.36, −0.34**
Governmental hospital (ref. no)	–	–	–	–	–	–	–	–	0.35	0.25	0.157	−0.14, 0.84
Private hospital or clinic (ref. no)	**−1.57**	**0.51**	**0.002**	**−2.58, −0.56**	–	–	–	–	−0.38	0.24	0.115	−0.86, 0.09
More than one dispute (ref. one)	–	–	–	–	–	–	–	–	**0.67**	**0.23**	**0.004**	**0.21, 1.13**
Formal complaint (ref. lawsuit)	−0.92	0.56	0.101	−2.03, 0.18	–	–	–	–	**−0.64**	**0.24**	**0.008**	**−1.12, −0.17**
Threat or blackmail (ref. lawsuit)	0.32	0.93	0.732	−1.51, 2.15	–	–	–	–	0.23	0.40	0.569	−0.56, 1.02
Management-related allegation (ref. no)	0.93	0.53	0.081	−0.11, 1.97	**0.17**	**0.07**	**0.023**	**0.02, 0.31**	−0.00	0.24	0.991	−0.48, 0.48
Procedure-related allegation (ref. no)	–	–	–	–	–	–	–	–	0.31	0.26	0.222	−0.19, 0.82
Diagnosis-related allegation (ref. no)	**1.41**	**0.57**	**0.013**	**0.29, 2.52**	**0.25**	**0.08**	**0.001**	**0.10, 0.40**	–	–	–	–
Medication-related allegation (ref. no)	0.61	0.96	0.528	−1.28, 2.49	0.20	0.13	0.122	−0.05, 0.46	0.62	0.41	0.135	−0.19, 1.44
Unfavorable outcome (ref. favorable)	0.69	0.65	0.296	−0.60, 1.97	0.15	0.09	0.096	−0.03, 0.32	0.27	0.28	0.337	−0.29, 0.83
Still in progress (ref. favorable outcome)	0.36	0.72	0.619	−1.06, 1.77	0.01	0.10	0.917	−0.18, 0.20	−0.16	0.31	0.594	−0.77, 0.44
Personal support (ref. no)	**1.27**	**0.60**	**0.035**	**0.09, 2.46**	0.13	0.08	0.106	−0.03, 0.29	–	–	–	–
Workplace support (ref. no)	–	–	–	–	–	–	–	–	**−0.53**	**0.25**	**0.033**	**−1.02, −0.04**
Professional support (ref. no)	**2.36**	**0.57**	**<0.001**	**1.25, 3.47**	**0.30**	**0.07**	**<0.001**	**0.15, 0.45**	0.22	0.25	0.368	−0.26, 0.70
Constant	13.17				2.20				5.22			
R^2^; adjusted R^2^	0.131; 0.100				0.125; 0.097				0.177; 0.146			
F (df1, df2); *p*	4.26 (20, 565); <0.001				4.51 (18, 567); <0.001				5.74 (21, 562); <0.001			
VIF range	1.05–3.34				1.03–3.32				1.06–1.98			
Maximum Cook’s distance	0.028				0.018				0.026			
Breusch–Pagan *p*	0.228				0.391				0.029			

B = unstandardized coefficient; CI = confidence interval; IES-6 = Impact of Event Scale-6; K6 = Kessler Psychological Distress Scale-6; MBBCh = Bachelor of Medicine and Bachelor of Surgery; PTSS = post-traumatic stress symptoms; ref. = reference category; SE = standard error; VIF = variance inflation factor. Each model includes all variables with *p* < 0.20 in the bivariate analysis (Tables S6 and S7); – = not entered in that model. Bold: *p* < 0.05. Two participants without a specified specialty were excluded from the somatic symptom model (n = 584).

**Table 7:** Multivariable linear regression analysis of sociodemographic, occupational, and dispute-related correlates associated with defensive medicine behaviors and malpractice fear among study participants (*N* = 586).

Correlate (Reference)	Defensive Medicine (DMBS, 12 Items)				Malpractice Fear Score			
	B	SE	*p*	95% CI	B	SE	*p*	95% CI
Age ≥ 43 years (ref. <43 years)	−0.23	0.59	0.701	−1.38, 0.93	−0.21	0.15	0.160	−0.50, 0.08
Female (ref. male)	–	–	–	–	0.15	0.16	0.348	−0.17, 0.47
Diploma or master’s degree (ref. MD/PhD/fellowship/board)	−0.13	0.67	0.846	−1.44, 1.18	–	–	–	–
MBBCh only (ref. MD/PhD/fellowship/board)	**4.24**	**2.09**	**0.043**	**0.14, 8.33**	–	–	–	–
Non-surgical specialty (ref. surgical)	−0.07	0.61	0.905	−1.27, 1.12	−0.12	0.16	0.456	−0.45, 0.20
Specialist (ref. consultant)	1.21	0.73	0.097	−0.22, 2.64	–	–	–	–
Resident (ref. consultant)	−2.67	1.68	0.112	−5.97, 0.62	–	–	–	–
Practice in another Arab country (ref. Egypt)	−0.42	0.63	0.506	−1.67, 0.82	−0.25	0.16	0.130	−0.56, 0.07
Governmental hospital (ref. no)	0.06	0.55	0.914	−1.02, 1.13	0.20	0.15	0.167	−0.08, 0.49
Other hospital # (ref. no)	–	–	–	–	0.36	0.29	0.217	−0.21, 0.92
More than one dispute (ref. one)	–	–	–	–	**0.42**	**0.15**	**0.006**	**0.12, 0.73**
Formal complaint (ref. lawsuit)	−1.08	0.59	0.065	−2.24, 0.07	−0.21	0.16	0.183	−0.52, 0.10
Threat or blackmail (ref. lawsuit)	−0.45	0.93	0.625	−2.28, 1.37	0.22	0.25	0.377	−0.27, 0.72
Procedure-related allegation (ref. no)	−0.79	0.59	0.176	−1.95, 0.36	0.12	0.16	0.448	−0.20, 0.44
Diagnosis-related allegation (ref. no)	−0.92	0.61	0.133	−2.11, 0.28	−0.21	0.17	0.210	−0.54, 0.12
Medication-related allegation (ref. no)	−0.71	1.01	0.480	−2.69, 1.27	**0.59**	**0.27**	**0.032**	**0.05, 1.12**
Unfavorable outcome (ref. favorable)	0.07	0.69	0.922	−1.29, 1.42	0.29	0.18	0.116	−0.07, 0.65
Still in progress (ref. favorable outcome)	−0.22	0.75	0.768	−1.69, 1.25	**0.60**	**0.20**	**0.003**	**0.20, 0.99**
Personal support (ref. no)	0.12	0.63	0.847	−1.11, 1.35	–	–	–	–
Workplace support (ref. no)	0.24	0.58	0.678	−0.90, 1.39	−0.20	0.16	0.210	−0.51, 0.11
Psychological distress (K6 score)	0.09	0.06	0.161	−0.04, 0.21	**0.04**	**0.02**	**0.013**	**0.01, 0.08**
PTSS (IES-6 mean item score)	**0.93**	**0.46**	**0.045**	**0.02, 1.84**	**0.46**	**0.12**	**<0.001**	**0.22, 0.70**
Malpractice fear score	**3.32**	**0.15**	**<0.001**	**3.02, 3.63**	–	–	–	–
Constant	15.81				6.18			
R^2^; adjusted R^2^	0.566; 0.551				0.207; 0.183			
F (df1, df2); *p*	36.78 (20, 563); <0.001				8.69 (17, 566); <0.001			
VIF range	1.06–2.43				1.04–2.35			
Maximum Cook’s distance	0.046				0.032			
Breusch–Pagan *p*	0.169				<0.001			

B = unstandardized coefficient; CI = confidence interval; DMBS = Defensive Medicine Behavior Scale; IES-6 = Impact of Event Scale-6; K6 = Kessler Psychological Distress Scale-6; MBBCh = Bachelor of Medicine and Bachelor of Surgery; PTSS = post-traumatic stress symptoms; ref. = reference category; SE = standard error; VIF = variance inflation factor. Each model includes all variables with *p* < 0.20 in the bivariate analysis (Tables S8 and S9); – = not entered in that model. Bold: *p* < 0.05. Two participants without a specified specialty were excluded from both models (n = 584). The DMBS model without the malpractice fear score is shown in Table S11. # Hospitals affiliated with specialized bodies (Military Hospitals, General Authority for Health Insurance, Suez Canal Authority).

## Data Availability

The data presented in this study are available on reasonable request from the corresponding author. The data are not publicly available because of ethical restrictions that protect participants’ confidentiality.

## Supplementary Materials

The following supporting information can be downloaded at: https://www.mdpi.com/article/10.3390/healthcare14213657/s1, Table S1: Specialties of participating physicians (n = 586); Table S2: Distribution of the nature of alleged negligence in malpractice disputes among participating physicians (n = 586); Table S3: Bivariate analysis of sociodemographic and occupational factors associated with the nature of alleged negligence of malpractice disputes among study participants (n = 586); Table S4: Bivariate analysis of sociodemographic, occupational, and dispute-related factors associated with an unfavorable versus a favorable legal outcome among resolved malpractice disputes (n = 486); Table S5: Defensive medicine behaviors and malpractice fear associated with medical malpractice disputes among participating physicians (n = 586); Table S6: Bivariate analysis of sociodemographic and occupational factors associated with psychological and somatic burden of malpractice disputes among study participants (n = 586); Table S7: Bivariate analysis of dispute-related factors associated with psychological and somatic burdens of malpractice disputes among study participants (n = 586); Table S8: Bivariate analysis of sociodemographic and occupational factors associated with defensive medicine behaviors and malpractice fear among study participants (n = 586); Table S9: Bivariate analysis of dispute-related characteristics associated with defensive medicine behaviors and malpractice fear among study participants (n = 586); Table S10: Sensitivity analysis of legal outcomes with disputes still in progress counted as non-events (n = 586); Table S11: Sensitivity analysis of defensive medicine behavior without the malpractice fear score (n = 586); Table S12: Sensitivity analysis adjusted for specialty group (n = 586); File S1: Study questionnaire; File S2: STROBE checklist.

## Abbreviations

The following abbreviations are used in this manuscript:

AOR	Adjusted odds ratio
CI	Confidence interval
COR	Crude odds ratio
DMBS	Defensive Medicine Behavior Scale
IES-6	Impact of Event Scale-6
IQR	Interquartile range
K6	Kessler Psychological Distress Scale-6
MBBCh	Bachelor of Medicine and Bachelor of Surgery
MFS	Malpractice Fear Scale
MD	Doctor of Medicine (Egyptian postgraduate clinical doctorate)
PhD	Doctor of Philosophy
PTSS	Posttraumatic stress symptoms
SD	Standard deviation
SE	Standard error
VIF	Variance inflation factor
