# Supplementary material for: Nature, Characteristics, and Reported Experiences of Medical Malpractice Disputes Among Egyptian Physicians Practicing in Egypt and Other Arab Countries: A Cross-Sectional Medicolegal Analysis of Psychological Distress, Somatic Symptoms, and Defensive Behavior
Source: Healthcare (Basel). 2026 Oct 28;14(21):3657. doi: 10.3390/healthcare14213657 (PMC13763266; doi:10.3390/healthcare14213657)
Supplement: Supplementary file 1 [file healthcare-14-03657-s001.zip › File S1 Study questionnaire.pdf]

Nature, Characteristics, and Reported Experiences of Medical Malpractice Claims Among Egyptian Physicians: A Cross-Sectional Medico-legal Analysis of Psychological Distress, Somatic Symptoms, and Defensive Behavior

- This study aimed to investigate the nature and the psychological and physical impacts of medical malpractice allegations or lawsuits on the accused physicians.
- The protocol of this research has been approved by the Research Ethics Committee of the Faculty of Medicine, Alexandria University (IRB Number: 00012098, FWA Number: 00018699, Serial Protocol Number: 0306388).
- Any information or opinion that appears in this questionnaire will be completely confidential and used only for research purposes. Please answer freely without constraints.
- Participation in this survey is entirely voluntary. By completing and submitting this questionnaire, you indicate that you have read the study information above and give your informed consent to participate and for your responses to be used for research purposes.
- Please take your time filling out the questionnaire; accuracy of response is of much importance.

Thanks in advance.

1. I agree to participate in this research *
   - € Yes
2. I faced a medical malpractice claim at least once *
   - € Yes

I. Section I: Sociodemographic and Occupational Characteristics

3. Age: ……………………………………………
4. Gender
   - € Male
   - € Female
5. Marital status
   - € Single
   - € Married
   - € Divorced
   - € Widow
6. Highest degree in education
   - o MBBCh
   - o Diploma
   - o Masters
   - o MD/PhD

- Fellowship/Board

7. Specialty (Alphabetic order)
    - Andrology
    - Anesthesia, ICU
    - Audio vestibular medicine
    - Bariatric surgery
    - Cardiology
    - Cardiothoracic surgery
    - Clinical Oncology and nuclear medicine
    - Clinical pathology
    - Clinical toxicology
    - Colorectal surgery
    - Critical care medicine
    - Dermatology
    - Diagnostic and interventional radiology
    - Emergency medicine
    - Endocrinology
    - Gastroenterology and hepatology
    - General surgery
    - Geriatrics and gerontology
    - GIT surgery
    - Head and neck surgery
    - Hematology
    - Hepatobiliary surgery
    - Internal medicine
    - Medical oncology
    - Nephrology
    - Neurology
    - Neurosurgery
    - Obstetrics and Gynecology
    - Ophthalmology
    - Orthopedic surgery
    - Otorhinolaryngology
    - Pediatric surgery
    - Pediatrics
    - Phoniatrics
    - Plastic surgery

- o Psychiatry
- o Pulmonary medicine
- o Rheumatology and rehabilitation
- o Surgical oncology
- o Tropical medicine
- o Urology
- o Vascular surgery
- o Other (please specify)

8. Current Position
   - o Resident
   - o Specialist
   - o Consultant
9. Duration of employment (years) ………………………
10. Workplace (Choose all that apply)
    - € Governmental hospital (MOH)
    - € University Hospital
    - € Private Hospital
    - € Military Hospital
    - € Other……………………….
11. Country of practice
    - o Egypt
    - o Arab Country

II. Section II: Nature and Outcome of Medical Malpractice Disputes:

12. How many times have you faced medical malpractice disputes?
    - o Once
    - o Twice
    - o Other

Regarding the most recent dispute;

13. Type of disputes:
    - o Threatening
    - o Blackmailing
    - o Formal complaint
    - o Lawsuit
14. What was the nature of the alleged negligence? (Choose all that apply.)
    - € Misdiagnosis
    - € Non-detection of a lesion

- € Misinterpretation of a finding
- € Delayed diagnosis
- € Lack of communication of a diagnosis/finding with the patient.
- € Breach of confidentiality
- € Failure of/delayed referral.
- € Unethical conduct (e.g., examining a patient without a nurse's presence).
- € Incompetent physician (without the required qualifications)
- € Improper supervision of other staff personnel
- € Failure to supervise or monitor care
- € Failure to recognize a complication of treatment/surgery
- € Improper management of the treatment plan
- € Not indicated surgery
- € Surgical procedure not done or incomplete
- € Improper performance of the surgical procedure
- € Surgery in a non-equipped place
- € Inadequate postoperative follow-up
- € Operating on the wrong body part
- € Medication errors (incorrect dose or combination, improper formula or duration).
- € Failure to recognize and manage a side effect of treatment
- € Informed consent issue
- € Technical error with machinery
- € Complication during performance of a procedure (IV contrast allergic reaction in CT, IUD uterine perforation).
- € A surgical foreign body/towel was left in the patient
- € Faulty anesthetic procedure
- € Medical misadventure (unexpected patient injury outside of your control).

15. What was the harm severity associated with the claim?
    - o Patient's Death
    - o Patient's Permanent disability (loss of organ/function)
    - o Patient's Temporary disability
    - o Patient's/Family Emotional distress.
16. What was the legal outcome?
    - o Dropped before the court
    - o Settled with a payout to the plaintiff
    - o Went to trial with an innocence verdict
    - o Went to trial with a fine verdict

- o Went to trial with a prison sentence
- o Went to trial, still in progress
- o Administrative punishment

17. During the dispute, from whom did you receive support? (Check all that apply).
    - € Family.
    - € Friends.
    - € Colleagues.
    - € Supervisors/seniors.
    - € Hospital management.
    - € Syndicate
    - € Legal consultant/lawyer.
    - € Other:

III. Section III: Impact of Medical Malpractice Disputes

*A. **Psychological impact:***

18. During the process of the most recent medical malpractice dispute, how often did you feel

	All the time	Most of the time	Some of the time	A little of the time	None of the time
Nervous					
Hopeless					
Restless or fidgety					
Everything was an effort.					
So sad that nothing could cheer you up.					
Worthless.					

19. How frequently were these comments true for you during the process of the most recent medical malpractice dispute?

	Almost always	Often	Sometimes	Seldom	Never
I tried not to think about the dispute					
Other things kept making me think about the dispute.					
I thought about the dispute when I did not mean to					
I felt watchful or on guard.					
I had trouble concentrating.					
I was aware that I still had feelings about the dispute, but I didn't deal with them.					

*B. **Physical impact***

20. Did you experience or feel any of the following during the process of the most recent medical malpractice dispute?

	No	Yes
Changes in appetite or eating habits		
Headache		
Mental block		

Fatigue/reduced energy levels		
Insomnia or poor-quality sleep		
Exacerbation of previously diagnosed medical condition (diabetes, hypertension,…) (Symptoms are different or worse)		
Diarrhea		
Bronchial asthma		
Immunosuppression (recurrent infections)		
Dermatological diseases		
Angina/Myocardial infarction		
Arterial hypertension		
Digestive Hemorrhages		
Stroke		
Changes in libido (particularly reduction)		

C. Impact on practice

21. To protect myself from legal problems, I

	Strongly Agree	Agree	Neutral	Disagree	Strongly disagree
I order extra tests for my patients.					
I hospitalize patients for reasons other than indications					
I prescribe as many drugs as I can					
I spend more time with my patients.					
I explain medical procedures to my patients in more detail.					
I order more consultations on possible complications					
I use imaging techniques more often					
I keep more detailed records					
I place more emphasis on informed consent forms					
I prefer to use non-invasive protocols instead of interventional treatment protocols					
I avoid treatment protocols with high complication rates					
I avoid patients with complex medical problems.					
I avoid patients who are likely to sue					
I feel uncomfortable in practice, as malpractice is appearing more frequently in the media					
I changed the place where I practice					
I communicate better with staff					
I communicate better with patients.					
I become more attentive to patients' signals of doubt or high expectations of treatment					
I better safeguard my professional limits when following patients' wishes					
I became more careful when expressing opinions to patients regarding colleagues' work					

22. Indicate the extent to which you agree or disagree with the following statements.

	Strongly Agree	Agree	Neutral	Disagree	Strongly disagree
I have had to make significant changes in my practice pattern because of recent legal developments concerning medical delivery					
I am concerned I will be involved in a malpractice case sometime in the next 10 years					

I feel pressured in my day-to-day practice by the threat of malpractice litigation					
I order some tests or consultations to avoid the appearance of malpractice					
Sometimes I ask for consultant opinions primarily to reduce my risk of being sued					
Relying on clinical judgment rather than on technology to make a diagnosis is becoming riskier from a medicolegal perspective					

D. Personal opinions:

23. If you are given the opportunity to revisit your career choice,

If you are given the opportunity to revisit your career choice	No	Not Sure	Yes
Would you consider changing careers or limiting your practice?			
Would you choose to become a physician again?			
Would you choose your specialty again?			
Would you recommend that your children pursue careers as physicians or in your specialty?			

24. What is your assessment of the legal process for malpractice? *

- Heavily weighted to the plaintiff
- Somewhat weighted to the plaintiff
- Fair
- Somewhat weighted to the defendant
- Heavily weighted to the defendant

25. Thank you so much for your cooperation. Please feel free to add any comments.
