# Supplementary material for: Nature, Characteristics, and Reported Experiences of Medical Malpractice Disputes Among Egyptian Physicians Practicing in Egypt and Other Arab Countries: A Cross-Sectional Medicolegal Analysis of Psychological Distress, Somatic Symptoms, and Defensive Behavior
Source: Healthcare (Basel). 2026 Oct 28;14(21):3657. doi: 10.3390/healthcare14213657 (PMC13763266; doi:10.3390/healthcare14213657)
Supplement: Supplementary file 1 [file healthcare-14-03657-s001.zip › File S2 STROBE checklist.pdf]

File S2. STROBE checklist for cross-sectional studies

Manuscript: Nature, Characteristics, and Reported Experiences of Medical Malpractice Disputes Among Egyptian Physicians Practicing in Egypt and Other Arab Countries: A Cross-Sectional Medicolegal Analysis of Psychological Distress, Somatic Symptoms, and Defensive Behavior

**Section/topic**	**Item**	**Recommendation**	**Where reported in the manuscript**
Title and abstract	1	(a) Indicate the study's design with a commonly used term in the title or the abstract. (b) Provide an informative and balanced summary of what was done and what was found.	Title ("Cross-Sectional"); Abstract, Methods (cross-sectional online survey, nonprobability sampling); Abstract, Results and Conclusions
Background/rationale	2	Explain the scientific background and rationale for the investigation being reported.	Section 1 (Introduction), paragraphs 1–6
Objectives	3	State specific objectives, including any prespecified hypotheses.	Section 1, last paragraph
Study design	4	Present key elements of study design early in the paper.	Section 2, Study design and setting
Setting	5	Describe the setting, locations, and relevant dates, including periods of recruitment, exposure, follow-up, and data collection.	Section 2, Study design and setting (March–December 2025); Data collection and sampling strategy (online recruitment in Egypt and other Arab countries; dates of submissions)
Participants	6	(a) Give the eligibility criteria, and the sources and methods of selection of participants.	Section 2, Study population; Data collection and sampling strategy (screening questions, recruitment channels, removal of duplicate submissions)
Variables	7	Clearly define all outcomes, exposures, predictors, potential confounders, and effect modifiers. Give diagnostic criteria, if applicable.	Section 2, Study tool, items 1–8, and the definition paragraphs for dispute type, negligence, allegation coding, and medical and legal outcomes
Data sources/measurement	8	For each variable of interest, give sources of data and details of methods of assessment (measurement).	Section 2, Study tool, items 4–6 (scales, items included, scoring, validation, internal consistency, recall reference); File S1 (questionnaire)
Bias	9	Describe any efforts to address potential sources of bias.	Section 2, Data collection and sampling strategy; Section 4, Strengths and Limitations
Study size	10	Explain how the study size was arrived at.	Section 2, Sample size
Quantitative variables	11	Explain how quantitative variables were handled in the analyses. If applicable, describe which groupings were chosen and why.	Section 2, Study tool, items 4–6 (cut-offs and their basis); Statistical analysis (continuous scores in regression models)
Statistical methods	12	(a) Describe all statistical methods, including those used to control for confounding. (b) Describe any methods used to examine subgroups and interactions. (c) Explain how missing data were addressed. (d) If applicable, describe analytical methods taking account of sampling strategy. (e) Describe any sensitivity analyses.	(a) Section 2, Statistical analysis; (b) not performed; (c) specialty was not specified by two participants, who were excluded from analyses that included specialty; no other missing data (Statistical analysis); (d) nonprobability sample, no weighting (Data collection and sampling strategy); (e) pending disputes counted as non-events (Table S10), defensive medicine model without malpractice fear (Table S11), and adjustment for specialty group (Table S12);

			heteroscedasticity-robust standard errors and a stated robustness criterion (Statistical analysis; Section 3.7)
Participants	13	(a) Report numbers of individuals at each stage of study. (b) Give reasons for non-participation at each stage. (c) Consider use of a flow diagram.	(a) Section 2, Sample size and Data collection (588 submissions, 2 duplicates removed, 586 analyzed; 486 resolved disputes in the legal-outcome model, 484 with specialty recorded); (b) number approached and response rate could not be determined (Data collection and sampling strategy); (c) not applicable
Descriptive data	14	(a) Give characteristics of study participants and information on exposures and potential confounders. (b) Indicate number of participants with missing data for each variable of interest.	(a) Section 3.1, Table 1, Table S1; (b) specialty not specified by two participants (Table 1); no other missing data
Outcome data	15	Report numbers of outcome events or summary measures.	Section 3.2 and 3.4; Tables 2 and 5; Tables S2 and S5
Main results	16	(a) Give unadjusted estimates and, if applicable, confounder-adjusted estimates and their precision (e.g., 95% confidence interval). Make clear which confounders were adjusted for and why they were included. (b) Report category boundaries when continuous variables were categorized. (c) If relevant, consider translating estimates of relative risk into absolute risk.	(a) Unadjusted: Tables S3, S4, S6–S9; adjusted: Tables 3, 4, 6, and 7, which show every covariate entered; covariate selection in Statistical analysis. (b) Table 1 and Section 2 (age, employment duration, scale categories). (c) Not applicable
Other analyses	17	Report other analyses done, e.g., analyses of subgroups and interactions, and sensitivity analyses.	Section 3.3, paragraph 3; Section 3.7; Tables S10–S12
Key results	18	Summarize key results with reference to study objectives.	Section 4, Principal Findings
Limitations	19	Discuss limitations of the study, taking into account sources of potential bias or imprecision. Discuss both direction and magnitude of any potential bias.	Section 4, Strengths and Limitations
Interpretation	20	Give a cautious overall interpretation of results considering objectives, limitations, multiplicity of analyses, results from similar studies, and other relevant evidence.	Section 4; Section 5 (Conclusions)
Generalisability	21	Discuss the generalisability (external validity) of the study results.	Section 4, Strengths and Limitations (second, fifth, and sixth limitations, and specialty mix)
Funding	22	Give the source of funding and the role of the funders for the present study and, if applicable, for the original study on which the present article is based.	Funding statement (no external funding)

Locations refer to section and paragraph headings of the revised manuscript.
