# Supplementary material for: Nature, Characteristics, and Reported Experiences of Medical Malpractice Disputes Among Egyptian Physicians Practicing in Egypt and Other Arab Countries: A Cross-Sectional Medicolegal Analysis of Psychological Distress, Somatic Symptoms, and Defensive Behavior
Source: Healthcare (Basel). 2026 Oct 28;14(21):3657. doi: 10.3390/healthcare14213657 (PMC13763266; doi:10.3390/healthcare14213657)
Supplement: Supplementary file 1 [file healthcare-14-03657-s001.zip › Supplementary Tables.pdf]

Table S1. Specialties of participating physicians (n = 586).

Specialty	n (%)
Obstetrics and Gynecology	193 (32.9)
Ophthalmology	43 (7.3)
Orthopedic surgery	43 (7.3)
General surgery	40 (6.8)
Anesthesia, ICU	35 (6.0)
Pediatrics	32 (5.5)
Diagnostic and interventional radiology	18 (3.1)
Plastic surgery	18 (3.1)
Urology	14 (2.4)
Dermatology	13 (2.2)
Dentistry	12 (2.0)
Neurosurgery	12 (2.0)
Internal medicine	11 (1.9)
Otorhinolaryngology	11 (1.9)
Cardiology	9 (1.5)
Cardiothoracic surgery	8 (1.4)
Pulmonary medicine	8 (1.4)
Emergency medicine	7 (1.2)
Gastroenterology and hepatology	7 (1.2)
Pediatric surgery	6 (1.0)
Nephrology	5 (0.9)
Bariatric surgery	4 (0.7)
Clinical pathology	4 (0.7)
Medical oncology	4 (0.7)
Pathology	4 (0.7)
Vascular surgery	4 (0.7)
Clinical toxicology	3 (0.5)
Neurology	3 (0.5)
Surgical oncology	3 (0.5)
Critical care medicine	2 (0.3)
GIT surgery	2 (0.3)
Not specified	2 (0.3)
Psychiatry	2 (0.3)
Colorectal surgery	1 (0.2)
Family medicine	1 (0.2)
Hepatobiliary surgery	1 (0.2)
Tropical medicine	1 (0.2)

n = number. The surgical group (n = 415) comprised obstetrics and gynecology, ophthalmology, orthopedic surgery, general surgery, plastic surgery, urology, dentistry, neurosurgery, otorhinolaryngology, cardiothoracic surgery, pediatric surgery, bariatric surgery, vascular surgery, surgical oncology, gastrointestinal surgery, colorectal surgery, and hepatobiliary surgery; all other specialties formed the non-surgical group (n = 169), and two participants did not specify their specialty. Twelve participants (2.0%) were dentists.

Table S2. Distribution of the nature of alleged negligence in malpractice disputes among participating physicians (n = 586).

Allegation category	Operational definition and sorting logic	n (%) [a]
**I. Management-related**	**Failures in care planning, oversight, and professional or ethical stewardship.**	**348 (59.4)**
A. Professional and ethical stewardship		
Breach of confidentiality	Transgression of patient privacy regulations.	8 (1.4)
Unethical conduct	Departures from the professional code of ethics.	9 (1.5)
Incompetent physician	Allegations regarding general professional capacity.	37 (6.3)
B. Clinical coordination and oversight		
Failure of or delayed referral	Untimely transition of care to a specialist.	43 (7.3)
Improper supervision of staff personnel	Deficits in institutional or team oversight.	22 (3.8)
Failure to supervise or monitor care	Inadequate ongoing review of clinical status.	64 (10.9)

Allegation category	Operational definition and sorting logic	n (%) [a]
Failure to recognize a treatment complication	Delayed identification of secondary adverse events.	92 (15.7)
Improper management of the treatment plan	Core deviations in non-surgical care pathways.	156 (26.6)
C. Improper management of a surgical patient		
A non-indicated surgery	Failure of surgical justification.	16 (2.7)
Surgical procedure not done or incomplete	Unexecuted or unfinished intended surgery.	30 (5.1)
Improper performance of a surgical procedure	Technical deviations during surgery.	90 (15.4)
Surgery in an unequipped place	Environmental or logistical management failure.	18 (3.1)
Inadequate postoperative follow-up	Monitoring failures specific to the surgical window.	41 (7.0)
Operating on the wrong body part	Critical site-verification protocol failure.	5 (0.9)
**II. Diagnosis-related**	**Cognitive or communicative errors during clinical evaluation.**	**197 (33.6)**
Misdiagnosis	Incorrect identification of pathology.	107 (18.3)
Non-detection of a lesion	Failure to locate physical or radiologic findings.	36 (6.1)
Misinterpretation of a finding	Flawed analysis of diagnostic data or laboratory results.	35 (6.0)
Delayed diagnosis	Failure to diagnose within an acceptable time frame.	59 (10.1)
Lack of diagnosis communication with the patient	Failure to inform the patient of findings.	38 (6.5)
**III. Medication-related**	**Errors specific to pharmacotherapy.**	**45 (7.7)**
Medication errors	Incorrect dose, combination, formula, or duration.	20 (3.4)
Failure to recognize a side effect of treatment	Missed detection of known adverse drug reactions.	30 (5.1)
**IV. Procedure-related**	**Events tied directly to invasive interventions.**	**206 (35.2)**
Informed consent issue	Alleged failure to secure valid legal permission before an intervention; grouped here because it was raised alongside physical procedural complications.	27 (4.6)
Technical error with machinery	Equipment or device-related failures during a procedure.	20 (3.4)
Complication during performance of a procedure	Intraoperative or intra-procedural adverse events.	60 (10.2)
Surgical foreign body left in the patient	Retained items after a procedure.	19 (3.2)
Faulty anesthetic procedure	Complications arising from anesthesia delivery.	41 (7.0)
Medical misadventure	Unexpected patient injury outside the physician’s control.	84 (14.3)

n = number. Each category reflects the allegation made by the complainant, not a proven finding of negligence. Confidentiality breaches, unethical conduct, and medical misadventure fall outside negligence in its strict legal sense but were raised within the same disputes.

[a] Answers are not mutually exclusive. A physician could select several items, and the category total counts physicians with at least one item in that category; for example, the 20 medication-error and 30 side-effect selections came from 45 physicians.

Table S3. Bivariate analysis of sociodemographic and occupational factors associated with the nature of alleged negligence in malpractice disputes among study participants (n = 586).

Factor	Total n	Management-related			Procedure-related			Diagnosis-related			Medication-related		
		n (%) [a]	p	COR (95% CI)	n (%) [a]	p	COR (95% CI)	n (%) [a]	p	COR (95% CI)	n (%) [a]	p	COR (95% CI)
**Age**													
< 43 years	292	169 (57.9)	–	ref.	111 (38.0)	–	ref.	93 (31.8)	–	ref.	17 (5.8)	–	ref.
≥ 43 years	294	179 (60.9)	0.459	1.13 (0.81–1.58)	95 (32.3)	0.148	0.78 (0.55–1.09)	104 (35.4)	0.366	1.17 (0.83–1.65)	28 (9.5)	0.092	1.70 (0.91–3.18)
**Sex**													
Male	416	253 (60.8)	–	ref.	142 (34.1)	–	ref.	125 (30.0)	–	ref.	25 (6.0)	–	ref.
Female	170	95 (55.9)	0.270	0.82 (0.57–1.17)	64 (37.6)	0.419	1.17 (0.80–1.69)	72 (42.4)	0.004	1.71 (1.18–2.47)	20 (11.8)	0.018	2.09 (1.12–3.87)
**Marital status**													
Married	515	302 (58.6)	–	ref.	182 (35.3)	–	ref.	169 (32.8)	–	ref.	39 (7.6)	–	ref.
Not married	71	46 (64.8)	0.323	1.30 (0.77–2.18)	24 (33.8)	0.799	0.93 (0.55–1.58)	28 (39.4)	0.268	1.33 (0.80–2.22)	6 (8.5)	0.795	1.13 (0.46–2.76)
**Highest education**			0.046			0.430			0.551			0.487	
MD/PhD/Fellowship/Board	248	154 (62.1)	–	ref.	84 (33.9)	–	ref.	78 (31.5)	–	ref.	21 (8.5)	–	ref.
Diploma/Master's	324	182 (56.2)	0.154	0.78 (0.56–1.10)	119 (36.7)	0.479	1.13 (0.80–1.60)	115 (35.5)	0.311	1.20 (0.84–1.70)	22 (6.8)	0.451	0.79 (0.42–1.47)
MBBCh	14	12 (85.7)	0.074	3.66 (0.80–16.72)	3 (21.4)	0.398	0.53 (0.14–1.96)	4 (28.6)	1.000	0.87 (0.27–2.87)	2 (14.3)	0.353	1.80 (0.38–8.59)
**Specialty**													
Surgical	415	246 (59.3)	–	ref.	155 (37.3)	–	ref.	117 (28.2)	–	ref.	26 (6.3)	–	ref.
Non-surgical	169	101 (59.8)	0.914	1.02 (0.71–1.47)	51 (30.2)	0.100	0.72 (0.49–1.06)	78 (46.2)	<0.001	2.18 (1.51–3.16)	19 (11.2)	0.041	1.90 (1.02–3.53)
**Current position**			0.410			0.102			0.009			0.793	
Consultant	289	174 (60.2)	–	ref.	109 (37.7)	–	ref.	80 (27.7)	–	ref.	20 (6.9)	–	ref.
Specialist	273	157 (57.5)	0.516	0.89 (0.64–1.25)	93 (34.1)	0.367	0.85 (0.60–1.21)	106 (38.8)	0.005	1.66 (1.16–2.36)	23 (8.4)	0.502	1.24 (0.66–2.31)
Resident	24	17 (70.8)	0.305	1.61 (0.65–3.99)	4 (16.7)	0.039	0.33 (0.11–0.99)	11 (45.8)	0.060	2.21 (0.95–5.14)	2 (8.3)	0.681	1.22 (0.27–5.57)
**Employment duration**			0.538			0.370			0.773			0.838	
≤ 10 years	114	67 (58.8)	–	ref.	34 (29.8)	–	ref.	35 (30.7)	–	ref.	9 (7.9)	–	ref.
11–15 years	150	83 (55.3)	0.576	0.87 (0.53–1.42)	60 (40.0)	0.087	1.57 (0.93–2.63)	54 (36.0)	0.367	1.27 (0.76–2.13)	9 (6.0)	0.545	0.74 (0.29–1.94)
16–20 years	159	101 (63.5)	0.426	1.22 (0.75–2.00)	57 (35.8)	0.298	1.31 (0.78–2.20)	51 (32.1)	0.810	1.07 (0.63–1.79)	13 (8.2)	0.933	1.04 (0.43–2.52)
> 20 years	163	97 (59.5)	0.902	1.03 (0.63–1.68)	55 (33.7)	0.492	1.20 (0.72–2.01)	57 (35.0)	0.458	1.21 (0.73–2.03)	14 (8.6)	0.837	1.10 (0.46–2.63)
**Country of practice**													
Egypt	370	212 (57.3)	–	ref.	158 (42.7)	–	ref.	90 (24.3)	–	ref.	30 (8.1)	–	ref.
Other Arab country	216	136 (63.0)	0.178	1.27 (0.90–1.79)	48 (22.2)	<0.001	0.38 (0.26–0.56)	107 (49.5)	<0.001	3.05 (2.14–4.36)	15 (6.9)	0.610	0.85 (0.44–1.61)
**Governmental hospital**													
No	275	178 (64.7)	–	ref.	87 (31.6)	–	ref.	100 (36.4)	–	ref.	27 (9.8)	–	ref.
Yes	311	170 (54.7)	0.013	0.66 (0.47–0.92)	119 (38.3)	0.094	1.34 (0.95–1.89)	97 (31.2)	0.186	0.79 (0.56–1.12)	18 (5.8)	0.067	0.56 (0.30–1.05)
**Private hospital or clinic**													
No	294	171 (58.2)	–	ref.	96 (32.7)	–	ref.	98 (33.3)	–	ref.	19 (6.5)	–	ref.
Yes	292	177 (60.6)	0.545	1.11 (0.80–1.54)	110 (37.7)	0.203	1.25 (0.89–1.75)	99 (33.9)	0.884	1.03 (0.73–1.45)	26 (8.9)	0.267	1.41 (0.76–2.62)
**University hospital**													
No	478	280 (58.6)	–	ref.	156 (32.6)	–	ref.	173 (36.2)	–	ref.	32 (6.7)	–	ref.
Yes	108	68 (63.0)	0.402	1.20 (0.78–1.85)	50 (46.3)	0.007	1.78 (1.16–2.72)	24 (22.2)	0.006	0.50 (0.31–0.82)	13 (12.0)	0.060	1.91 (0.96–3.77)
**Other hospital #**													
No	547	331 (60.5)	–	ref.	185 (33.8)	–	ref.	190 (34.7)	–	ref.	43 (7.9)	–	ref.
Yes	39	17 (43.6)	0.038	0.50 (0.26–0.97)	21 (53.8)	0.011	2.28 (1.19–4.39)	7 (17.9)	0.032	0.41 (0.18–0.95)	2 (5.1)	0.759	0.63 (0.15–2.72)

CI = confidence interval; COR = crude odds ratio; MBBCh = Bachelor of Medicine and Bachelor of Surgery; n = number; ref. = reference category. MD/PhD/fellowship/board = MD, PhD, fellowship, or board certification. Specialty was not specified by two participants, who are excluded from the specialty comparison.

[a] Row percentages. Answers are not mutually exclusive. p values compare each group with the reference group (chi-squared test, or Fisher's exact test when an expected count was below 5); for factors with three or more groups, the p value in the factor row is from the overall chi-squared test, which was used to select variables for the multivariable models ($p < 0.20$).

Hospitals affiliated with specialized bodies (Military Hospitals, General Authority for Health Insurance, Suez Canal Authority).

Table S4. Bivariate analysis of sociodemographic, occupational, and dispute-related factors associated with an unfavorable versus a favorable legal outcome among resolved malpractice disputes (n = 486).

Factor	Total n	Unfavorable (n = 125) versus favorable outcome (n = 361)		
		n (%) [a]	p	COR (95% CI)
**Age**				
< 43 years	232	60 (25.9)	–	ref.
≥ 43 years	254	65 (25.6)	0.945	0.99 (0.66–1.48)
**Sex**				
Male	340	84 (24.7)	–	ref.
Female	146	41 (28.1)	0.435	1.19 (0.77–1.84)
**Marital status**				
Married	422	107 (25.4)	–	ref.
Not married	64	18 (28.1)	0.637	1.15 (0.64–2.07)
**Highest education**			0.070	
MD/PhD/Fellowship/Board	202	41 (20.3)	–	ref.
Diploma/Master’s	274	81 (29.6)	0.022	1.65 (1.07–2.53)
MBBCh	10	3 (30.0)	0.436	1.68 (0.42–6.79)
**Specialty**				
Surgical	347	98 (28.2)	–	ref.
Non-surgical	137	26 (19.0)	0.035	0.60 (0.37–0.97)
**Current position**			0.012	
Consultant	246	49 (19.9)	–	ref.
Specialist	220	70 (31.8)	0.003	1.88 (1.23–2.86)
Resident	20	6 (30.0)	0.265	1.72 (0.63–4.71)
**Employment duration**			0.229	
≤ 10 years	88	28 (31.8)	–	ref.
11–15 years	128	33 (25.8)	0.333	0.74 (0.41–1.35)
16–20 years	127	25 (19.7)	0.042	0.53 (0.28–0.98)
> 20 years	143	39 (27.3)	0.460	0.80 (0.45–1.44)
**Country of practice**				
Egypt	323	82 (25.4)	–	ref.
Other Arab country	163	43 (26.4)	0.813	1.05 (0.69–1.62)
**Governmental hospital**				
No	217	52 (24.0)	–	ref.
Yes	269	73 (27.1)	0.426	1.18 (0.78–1.78)
**Private hospital or clinic**				
No	252	59 (23.4)	–	ref.
Yes	234	66 (28.2)	0.227	1.29 (0.85–1.93)
**University hospital**				
No	394	108 (27.4)	–	ref.
Yes	92	17 (18.5)	0.078	0.60 (0.34–1.06)
**Other hospital #**				
No	452	116 (25.7)	–	ref.
Yes	34	9 (26.5)	0.917	1.04 (0.47–2.30)
**Number of disputes**				
One	288	64 (22.2)	–	ref.
More than one	198	61 (30.8)	0.033	1.56 (1.03–2.35)
**Dispute type**			<0.001	
Lawsuit	236	84 (35.6)	–	ref.

Factor	Total n	Unfavorable (n = 125) versus favorable outcome (n = 361)		
		n (%) [a]	p	COR (95% CI)
Formal complaint	190	27 (14.2)	<0.001	0.30 (0.18–0.49)
Threat or blackmail	60	14 (23.3)	0.072	0.55 (0.29–1.06)
**Management-related allegation**				
No	196	49 (25.0)	–	ref.
Yes	290	76 (26.2)	0.765	1.07 (0.70–1.62)
**Procedure-related allegation**				
No	314	71 (22.6)	–	ref.
Yes	172	54 (31.4)	0.034	1.57 (1.03–2.38)
**Diagnosis-related allegation**				
No	327	89 (27.2)	–	ref.
Yes	159	36 (22.6)	0.279	0.78 (0.50–1.22)
**Medication-related allegation**				
No	448	113 (25.2)	–	ref.
Yes	38	12 (31.6)	0.389	1.37 (0.67–2.80)
**Medical outcome**			0.257	
Emotional distress	225	50 (22.2)	–	ref.
Disability	145	41 (28.3)	0.187	1.38 (0.85–2.23)
Death	116	34 (29.3)	0.150	1.45 (0.87–2.41)

CI = confidence interval; COR = crude odds ratio; MBBCh = Bachelor of Medicine and Bachelor of Surgery; n = number; ref. = reference category. Favorable and unfavorable outcomes are defined in the main text (Section 2) and are not equivalent to judicial acquittal and conviction. Specialty was not specified by two participants, who are excluded from the specialty comparison.
[a] Row percentages among the 486 resolved disputes; the 100 disputes still in progress were excluded. p values as in Table S3.
Hospitals affiliated with specialized bodies (Military Hospitals, General Authority for Health Insurance, Suez Canal Authority).

Table S5. Defensive medicine behaviors and malpractice fear associated with medical malpractice disputes among participating physicians (n = 586).

To protect myself from legal problems, I …	Strongly agree n (%)	Agree n (%)	Neutral n (%)	Disagree n (%)	Strongly disagree n (%)
**Defensive Medicine Behavior Scale (12 of 14 items) [a]**					
Positive (assurance) defensive medicine					
I order extra tests for my patients (item 1)	131 (22.4)	155 (26.5)	169 (28.8)	73 (12.5)	58 (9.9)
I hospitalize patients for reasons other than indications (item 2)	122 (20.8)	155 (26.5)	162 (27.6)	82 (14.0)	65 (11.1)
I prescribe as many drugs as I can (item 3)	161 (27.5)	185 (31.6)	169 (28.8)	50 (8.5)	21 (3.6)
I spend more time with my patients (item 4)	185 (31.6)	196 (33.4)	169 (28.8)	24 (4.1)	12 (2.0)
I explain medical procedures to my patients in more detail (item 5)	226 (38.6)	216 (36.9)	117 (20.0)	15 (2.6)	12 (2.0)
I order more consultations on possible complications (item 6)	200 (34.1)	194 (33.1)	145 (24.7)	36 (6.1)	11 (1.9)
I keep more detailed records (item 8)	264 (45.1)	183 (31.2)	118 (20.1)	13 (2.2)	8 (1.4)
Negative (avoidance) defensive medicine					
I prefer non-invasive protocols instead of interventional treatment protocols (item 10)	175 (29.9)	167 (28.5)	172 (29.4)	46 (7.8)	26 (4.4)
I avoid treatment protocols with high complication rates (item 11)	245 (41.8)	165 (28.2)	124 (21.2)	34 (5.8)	18 (3.1)
I avoid patients with complex medical problems (item 12)	249 (42.5)	152 (25.9)	131 (22.4)	31 (5.3)	23 (3.9)
I avoid patients who are likely to sue (item 13)	282 (48.1)	144 (24.6)	122 (20.8)	21 (3.6)	17 (2.9)
I feel uncomfortable in practice, as malpractice is appearing more frequently in the media (item 14)	211 (36.0)	177 (30.2)	138 (23.5)	27 (4.6)	33 (5.6)
**Malpractice Fear Scale (2 of 6 items) [b]**					
I am concerned that I will be involved in a malpractice case at any time	244 (41.6)	185 (31.6)	128 (21.8)	16 (2.7)	13 (2.2)
I feel pressured in my day-to-day practice by the threat of malpractice litigation	252 (43.0)	176 (30.0)	120 (20.5)	24 (4.1)	14 (2.4)

n = number.

[a] Defensive Medicine Behavior Scale (DMBS). Items 1–6 and 8 form the positive (assurance) subscale and items 10–14 the negative (avoidance) subscale. Items 7 (“I use imaging techniques more often”) and 9 (“I place more emphasis on informed consent forms”) were not part of the questionnaire.

[b] Two of the six items of the Malpractice Fear Scale; their sum forms the malpractice fear score (range 2–10).

Table S6. Bivariate analysis of sociodemographic and occupational factors associated with psychological and somatic burden of malpractice disputes among study participants (n = 586).

Factor	Psychological distress (K6)			PTSS (IES-6)			Somatic symptom score		
	Median (IQR)	Test statistic	p	Median (IQR)	Test statistic	p	Median (IQR)	Test statistic	p
**Age**		Z = −3.06	**0.002**		Z = −1.62	0.106		Z = −0.10	0.918
< 43 years (n = 292)	17.0 (9.8)			2.8 (1.3)			5.0 (3.0)		
≥ 43 years (n = 294)	15.0 (9.2)			2.5 (1.3)			5.0 (4.2)		
**Sex**		Z = 3.20	**0.001**		Z = 3.70	**<0.001**		Z = 3.44	**<0.001**
Male (n = 416)	15.0 (9.0)			2.5 (1.2)			5.0 (4.0)		
Female (n = 170)	18.0 (10.0)			3.0 (1.2)			5.0 (3.0)		
**Marital status**		Z = 0.80	0.425		Z = 1.91	0.056		Z = −1.79	0.074
Married (n = 515)	16.0 (10.0)			2.7 (1.3)			5.0 (4.0)		
Not married (n = 71)	16.0 (12.0)			3.0 (1.5)			4.0 (4.0)		
**Highest education**		H = 4.62	0.099		H = 6.50	**0.039**		H = 3.50	0.174
MD/PhD/Fellowship/Board (n = 248)	15.0 (9.0)			2.7 (1.3)			5.0 (4.0)		
Diploma/Master's (n = 324)	16.0 (9.0)			2.7 (1.3)			5.0 (4.0)		
MBBCh (n = 14)	21.0 (9.2)			3.2 (1.5)			5.0 (5.2)		
**Specialty**		Z = −0.43	0.667		Z = −0.42	0.676		Z = −2.50	**0.012**
Surgical (n = 415)	16.0 (10.0)			2.7 (1.3)			5.0 (4.0)		
Non-surgical (n = 169)	15.0 (10.0)			2.7 (1.3)			5.0 (4.0)		
**Current position**		H = 7.37	**0.025**		H = 13.61	**0.001**		H = 1.48	0.476
Consultant (n = 289)	15.0 (9.0)			2.5 (1.2)			5.0 (4.0)		
Specialist (n = 273)	17.0 (9.0)			2.8 (1.3)			5.0 (4.0)		
Resident (n = 24)	18.0 (10.8)			3.0 (1.1)			5.0 (4.0)		
**Employment duration**		H = 9.35	**0.025**		H = 5.34	0.148		H = 14.11	**0.003**
≤ 10 years (n = 114)	16.0 (9.0)			2.8 (1.4)			5.0 (3.0)		
11–15 years (n = 150)	18.0 (10.0)			2.8 (1.2)			5.0 (4.0)		
16–20 years (n = 159)	13.0 (8.0)			2.5 (1.2)			4.0 (4.0)		
> 20 years (n = 163)	15.0 (9.0)			2.7 (1.3)			5.0 (4.0)		
**Country of practice**		Z = −2.24	**0.025**		Z = −2.33	**0.020**		Z = −5.30	**<0.001**
Egypt (n = 370)	17.0 (9.0)			2.8 (1.3)			6.0 (4.0)		
Other Arab country (n = 216)	14.0 (10.0)			2.5 (1.2)			4.0 (4.0)		
**Governmental hospital**		Z = 1.06	0.290		Z = −0.16	0.875		Z = 3.60	**<0.001**
No (n = 275)	15.0 (11.0)			2.7 (1.5)			4.0 (4.0)		
Yes (n = 311)	17.0 (8.0)			2.7 (1.2)			5.0 (4.0)		
**Private hospital or clinic**		Z = −2.25	**0.024**		Z = −1.17	0.242		Z = −2.53	**0.011**
No (n = 294)	17.0 (9.0)			2.8 (1.0)			5.0 (4.0)		
Yes (n = 292)	15.0 (11.0)			2.5 (1.5)			5.0 (4.0)		
**University hospital**		Z = −0.93	0.351		Z = −1.02	0.306		Z = 0.16	0.873
No (n = 478)	16.0 (10.0)			2.7 (1.3)			5.0 (4.0)		
Yes (n = 108)	15.0 (9.8)			2.7 (1.3)			5.0 (4.0)		
**Other hospital #**		Z = 0.54	0.587		Z = 1.11	0.266		Z = 1.11	0.268
No (n = 547)	16.0 (10.0)			2.7 (1.3)			5.0 (4.0)		
Yes (n = 39)	16.0 (9.0)			2.8 (0.7)			5.0 (4.0)		

H = Kruskal–Wallis H; IQR = interquartile range; MBBCh = Bachelor of Medicine and Bachelor of Surgery; Z = standardized Mann–Whitney U. For factors with two groups, Z is positive when the second group has higher values. IES-6 = Impact of Event Scale-6; K6 = Kessler Psychological Distress Scale-6; PTSS = post-traumatic stress symptoms. K6 and IES-6 scores refer to the period of the most recent dispute. Specialty was not specified by two participants, who are excluded from the specialty comparison.

Hospitals affiliated with specialized bodies (Military Hospitals, General Authority for Health Insurance, Suez Canal Authority).

Table S7. Bivariate analysis of dispute-related factors associated with psychological and somatic burdens of malpractice disputes among study participants (n = 586).

Factor	Psychological distress (K6)			PTSS (IES-6)			Somatic symptom score		
	Median (IQR)	Test statistic	p	Median (IQR)	Test statistic	p	Median (IQR)	Test statistic	p
**Number of disputes**		Z = −0.75	0.454		Z = −0.16	0.871		Z = 4.51	**<0.001**
One (n = 359)	17.0 (10.0)			2.7 (1.3)			5.0 (4.0)		
More than one (n = 227)	15.0 (9.0)			2.7 (1.3)			6.0 (3.0)		
**Dispute type**		H = 3.65	0.161		H = 2.55	0.280		H = 15.77	**<0.001**
Lawsuit (n = 301)	17.0 (11.0)			2.8 (1.3)			5.0 (4.0)		
Formal complaint (n = 225)	15.0 (8.0)			2.5 (1.2)			5.0 (4.0)		
Threat or blackmail (n = 60)	16.5 (10.8)			2.8 (1.6)			6.0 (3.0)		
**Management-related allegation**		Z = 1.38	0.169		Z = 1.51	0.132		Z = −1.28	0.199
No (n = 238)	16.0 (9.2)			2.7 (1.2)			5.0 (4.0)		
Yes (n = 348)	17.0 (10.0)			2.8 (1.3)			5.0 (5.0)		
**Procedure-related allegation**		Z = 0.75	0.456		Z = −0.36	0.717		Z = 3.45	**<0.001**
No (n = 380)	15.5 (9.8)			2.7 (1.3)			5.0 (4.0)		
Yes (n = 206)	17.0 (10.0)			2.7 (1.3)			6.0 (4.0)		
**Diagnosis-related allegation**		Z = 2.06	**0.040**		Z = 2.49	**0.013**		Z = −0.21	0.835
No (n = 389)	16.0 (9.0)			2.7 (1.2)			5.0 (4.0)		
Yes (n = 197)	17.0 (10.0)			2.8 (1.5)			5.0 (3.0)		
**Medication-related allegation**		Z = 1.31	0.191		Z = 2.11	**0.035**		Z = 2.12	**0.034**
No (n = 541)	16.0 (9.5)			2.7 (1.2)			5.0 (4.0)		
Yes (n = 45)	18.0 (10.0)			3.0 (1.7)			6.0 (4.5)		
**Medical outcome**		H = 2.09	0.352		H = 0.65	0.722		H = 0.07	0.967
Emotional distress (n = 258)	15.5 (10.0)			2.7 (1.2)			5.0 (4.0)		
Disability (n = 185)	16.0 (9.5)			2.7 (1.2)			5.0 (4.0)		
Death (n = 143)	17.0 (9.0)			2.7 (1.5)			5.0 (3.0)		
**Legal outcome**		H = 3.76	0.153		H = 5.17	0.075		H = 11.51	**0.003**
Acquittal (n = 361)	16.0 (9.0)			2.7 (1.2)			5.0 (5.0)		
Conviction (n = 125)	17.0 (8.5)			2.8 (1.2)			6.0 (3.0)		
Still in progress (n = 100)	16.0 (11.5)			2.5 (1.3)			5.0 (3.8)		
**Personal support**		Z = 2.00	**0.046**		Z = 1.59	0.111		Z = −0.57	0.571
No (n = 140)	15.0 (9.5)			2.5 (1.1)			5.0 (4.0)		
Yes (n = 446)	16.5 (9.0)			2.7 (1.3)			5.0 (3.0)		
**Workplace support**		Z = −1.23	0.218		Z = −0.30	0.766		Z = −4.18	**<0.001**
No (n = 178)	16.5 (9.2)			2.7 (1.3)			6.0 (3.0)		
Yes (n = 408)	16.0 (9.0)			2.7 (1.3)			5.0 (4.0)		
**Professional support**		Z = 4.59	**<0.001**		Z = 4.38	**<0.001**		Z = 1.97	**0.049**
No (n = 338)	14.0 (9.2)			2.5 (1.2)			5.0 (4.0)		
Yes (n = 248)	18.0 (11.0)			3.0 (1.7)			5.0 (4.0)		

H = Kruskal–Wallis H; IQR = interquartile range; MBBCh = Bachelor of Medicine and Bachelor of Surgery; Z = standardized Mann–Whitney U. For factors with two groups, Z is positive when the second group has higher values. IES-6 = Impact of Event Scale-6; K6 = Kessler Psychological Distress Scale-6; PTSS = post-traumatic stress symptoms. Allegation categories and types of support are not mutually exclusive. K6 and IES-6 scores refer to the period of the most recent dispute.

Table S8. Bivariate analysis of sociodemographic and occupational factors associated with defensive medicine behaviors and malpractice fear among study participants (n = 586).

Factor	DMBS (12 items)			Malpractice fear score		
	Median (IQR)	Test statistic	p	Median (IQR)	Test statistic	p
**Age**		Z = −2.46	**0.014**		Z = −2.05	**0.041**
< 43 years (n = 292)	48.0 (11.8)			8.0 (2.0)		
≥ 43 years (n = 294)	45.5 (16.0)			8.0 (4.0)		
**Sex**		Z = −0.39	0.694		Z = 1.61	0.107
Male (n = 416)	48.0 (16.0)			8.0 (4.0)		
Female (n = 170)	46.5 (11.0)			8.0 (2.0)		
**Marital status**		Z = 0.15	0.880		Z = 0.49	0.622
Married (n = 515)	47.0 (14.0)			8.0 (3.0)		
Not married (n = 71)	48.0 (11.0)			8.0 (3.0)		
**Highest education**		H = 7.03	**0.030**		H = 3.07	0.216
MD/PhD/Fellowship/Board (n = 248)	45.0 (14.0)			8.0 (4.0)		
Diploma/Master's (n = 324)	48.0 (13.0)			8.0 (2.0)		
MBBCh (n = 14)	51.5 (15.0)			8.5 (3.2)		
**Specialty**		Z = −2.15	**0.031**		Z = −2.07	**0.038**
Surgical (n = 415)	48.0 (13.0)			8.0 (3.0)		
Non-surgical (n = 169)	46.0 (15.5)			8.0 (4.0)		
**Current position**		H = 8.26	**0.016**		H = 2.95	0.229
Consultant (n = 289)	45.0 (15.0)			8.0 (4.0)		
Specialist (n = 273)	48.0 (12.0)			8.0 (2.0)		
Resident (n = 24)	46.0 (14.5)			8.0 (4.0)		
**Employment duration**		H = 1.20	0.753		H = 2.12	0.547
≤ 10 years (n = 114)	46.0 (14.0)			8.5 (3.0)		
11–15 years (n = 150)	48.0 (10.0)			8.0 (3.0)		
16–20 years (n = 159)	46.0 (17.0)			8.0 (4.0)		
> 20 years (n = 163)	48.0 (15.0)			8.0 (4.0)		
**Country of practice**		Z = −2.93	**0.003**		Z = −3.67	**<0.001**
Egypt (n = 370)	48.0 (13.0)			9.0 (3.0)		
Other Arab country (n = 216)	45.0 (13.0)			8.0 (4.0)		
**Governmental hospital**		Z = 1.63	0.103		Z = 1.99	**0.047**
No (n = 275)	46.0 (15.0)			8.0 (4.0)		
Yes (n = 311)	48.0 (13.0)			8.0 (3.0)		
**Private hospital or clinic**		Z = 0.23	0.821		Z = 0.50	0.621
No (n = 294)	47.0 (14.0)			8.0 (3.0)		
Yes (n = 292)	48.0 (14.0)			8.0 (3.0)		
**University hospital**		Z = −0.60	0.552		Z = 0.65	0.517
No (n = 478)	48.0 (14.0)			8.0 (3.0)		
Yes (n = 108)	45.5 (12.0)			8.0 (3.0)		
**Other hospital #**		Z = 0.69	0.492		Z = 1.52	0.128
No (n = 547)	47.0 (14.0)			8.0 (3.0)		
Yes (n = 39)	48.0 (14.0)			9.0 (2.0)		

H = Kruskal–Wallis H; IQR = interquartile range; MBBCh = Bachelor of Medicine and Bachelor of Surgery; Z = standardized Mann–Whitney U. For factors with two groups, Z is positive when the second group has higher values. DMBS = Defensive Medicine Behavior Scale (12 items). Specialty was not specified by two participants, who are excluded from the specialty comparison.

Hospitals affiliated with specialized bodies (Military Hospitals, General Authority for Health Insurance, Suez Canal Authority).

Table S9. Bivariate analysis of dispute-related characteristics associated with defensive medicine behaviors and malpractice fear among study participants (n = 586).

Factor	DMBS (12 items)			Malpractice fear score		
	Median (IQR)	Test statistic	p	Median (IQR)	Test statistic	p
**Number of disputes**		Z = 1.18	0.239		Z = 3.60	**<0.001**
One (n = 359)	47.0 (16.0)			8.0 (4.0)		
More than one (n = 227)	48.0 (13.0)			9.0 (2.0)		
**Dispute type**		H = 15.12	**<0.001**		H = 9.80	**0.007**
Lawsuit (n = 301)	48.0 (13.0)			8.0 (3.0)		
Formal complaint (n = 225)	45.0 (14.5)			8.0 (4.0)		
Threat or blackmail (n = 60)	48.5 (12.0)			9.0 (2.0)		
**Management-related allegation**		Z = −0.17	0.864		Z = −0.90	0.368
No (n = 238)	47.0 (11.0)			8.0 (3.0)		
Yes (n = 348)	48.0 (17.0)			8.0 (4.0)		
**Procedure-related allegation**		Z = 1.92	0.054		Z = 3.39	**<0.001**
No (n = 380)	46.0 (16.8)			8.0 (4.0)		
Yes (n = 206)	48.0 (11.0)			9.0 (2.0)		
**Diagnosis-related allegation**		Z = −1.59	0.112		Z = −2.11	**0.035**
No (n = 389)	48.0 (14.0)			8.0 (3.0)		
Yes (n = 197)	46.0 (14.0)			8.0 (4.0)		
**Medication-related allegation**		Z = 1.72	0.086		Z = 2.88	**0.004**
No (n = 541)	47.0 (14.0)			8.0 (3.0)		
Yes (n = 45)	49.0 (15.5)			10.0 (2.0)		
**Medical outcome**		H = 3.02	0.221		H = 0.66	0.721
Emotional distress (n = 258)	48.0 (14.0)			8.0 (3.0)		
Disability (n = 185)	46.0 (14.0)			8.0 (4.0)		
Death (n = 143)	48.0 (14.0)			8.0 (3.0)		
**Legal outcome**		H = 12.14	**0.002**		H = 15.15	**<0.001**
Acquittal (n = 361)	46.0 (14.5)			8.0 (4.0)		
Conviction (n = 125)	49.0 (12.5)			9.0 (2.0)		
Still in progress (n = 100)	48.0 (14.5)			8.0 (2.0)		
**Personal support**		Z = 1.69	0.092		Z = 0.71	0.479
No (n = 140)	45.0 (14.8)			8.0 (4.0)		
Yes (n = 446)	48.0 (13.2)			8.0 (3.0)		
**Workplace support**		Z = −2.03	**0.042**		Z = −3.17	**0.002**
No (n = 178)	48.0 (12.0)			9.0 (2.0)		
Yes (n = 408)	46.0 (15.8)			8.0 (4.0)		
**Professional support**		Z = 0.56	0.579		Z = −0.32	0.746
No (n = 338)	47.0 (12.2)			8.0 (3.0)		
Yes (n = 248)	48.0 (18.0)			8.0 (4.0)		
**Malpractice fear score**	–	rho = 0.719	<0.001	–	–	–
**Psychological distress (K6)**	–	rho = 0.349	<0.001	–	rho = 0.318	<0.001
**PTSS (IES-6)**	–	rho = 0.354	<0.001	–	rho = 0.329	<0.001

H = Kruskal–Wallis H; IQR = interquartile range; MBBCh = Bachelor of Medicine and Bachelor of Surgery; Z = standardized Mann–Whitney U. For factors with two groups, Z is positive when the second group has higher values. DMBS = Defensive Medicine Behavior Scale (12 items); IES-6 = Impact of Event Scale-6; K6 = Kessler Psychological Distress Scale-6; PTSS = post-traumatic stress symptoms; rho = Spearman's rank correlation coefficient.

Table S10. Sensitivity analysis of legal outcomes with disputes still in progress counted as non-events (n = 586).

Correlate (reference)	Unfavorable outcome versus all others			Favorable outcome versus all others		
	B	p	AOR (95% CI)	B	p	AOR (95% CI)
Diploma or master's degree (ref. MD/PhD/fellowship/board)	0.13	0.632	1.14 (0.67–1.95)	0.26	0.262	1.29 (0.82–2.03)
MBBCh only (ref. MD/PhD/fellowship/board)	0.03	0.968	1.03 (0.21–5.03)	−0.38	0.582	0.69 (0.18–2.62)
Non-surgical specialty (ref. surgical)	−0.46	0.072	0.63 (0.38–1.04)	0.30	0.144	1.35 (0.90–2.01)
Specialist (ref. consultant)	0.50	0.060	1.65 (0.98–2.77)	**−0.71**	**0.002**	**0.49 (0.31–0.77)**
Resident (ref. consultant)	0.90	0.137	2.46 (0.75–8.04)	−0.65	0.235	0.52 (0.18–1.52)
University hospital (ref. no)	−0.53	0.107	0.59 (0.31–1.12)	0.49	0.062	1.64 (0.97–2.75)
More than one dispute (ref. one)	**0.48**	**0.029**	**1.62 (1.05–2.49)**	−0.02	0.913	0.98 (0.67–1.42)
Formal complaint (ref. lawsuit)	**−1.04**	**<0.001**	**0.35 (0.22–0.58)**	**1.01**	**<0.001**	**2.75 (1.87–4.06)**
Threat or blackmail (ref. lawsuit)	−0.42	0.225	0.66 (0.33–1.29)	**1.33**	**<0.001**	**3.79 (1.96–7.34)**
Procedure-related allegation (ref. no)	0.38	0.087	1.47 (0.95–2.27)	**−0.39**	**0.044**	**0.68 (0.47–0.99)**
**Constant**	−1.45			0.20		
**Model χ² (df); p**	44.9 (10); <0.001			56.2 (10); <0.001		
**Nagelkerke R²**	0.115			0.125		
**Correctly classified, %**	78.1			62.8		
**Hosmer–Lemeshow χ² (8 df); p**	6.03; 0.644			7.73; 0.460		
**VIF range**	1.05–1.62			1.05–1.62		
**Maximum Cook's distance**	0.042			0.022		
**n (events)**	584 (124)			584 (360)		

AOR = adjusted odds ratio; B = unstandardized coefficient; CI = confidence interval; MBBCh = Bachelor of Medicine and Bachelor of Surgery; ref. = reference category; VIF = variance inflation factor. Both models include the same variables as the main model in Table 4. An unfavorable outcome (n = 125) was compared with a favorable outcome or a pending dispute (n = 461), and a favorable outcome (n = 361) with an unfavorable outcome or a pending dispute (n = 225). Two participants without a specified specialty were excluded from both models. Bold: $p < 0.05$.

Table S11. Sensitivity analysis of defensive medicine behavior without the malpractice fear score (n = 586).

Correlate (reference)	With malpractice fear score (Table 7)				Without malpractice fear score			
	B	SE	p	95% CI	B	SE	p	95% CI
Age ≥ 43 years (ref. < 43 years)	−0.23	0.59	0.701	−1.38, 0.93	−0.80	0.79	0.315	−2.36, 0.76
Diploma or master's degree (ref. MD/PhD/fellowship/board)	−0.13	0.67	0.846	−1.44, 1.18	0.46	0.90	0.610	−1.31, 2.22
MBBCh only (ref. MD/PhD/fellowship/board)	**4.24**	**2.09**	**0.043**	**0.14, 8.33**	5.30	2.82	0.060	−0.23, 10.83
Non-surgical specialty (ref. surgical)	−0.07	0.61	0.905	−1.27, 1.12	−0.57	0.82	0.492	−2.18, 1.05
Specialist (ref. consultant)	1.21	0.73	0.097	−0.22, 2.64	1.35	0.98	0.170	−0.58, 3.28
Resident (ref. consultant)	−2.67	1.68	0.112	−5.97, 0.62	**−4.67**	**2.26**	**0.040**	**−9.12, −0.22**
Practice in another Arab country (ref. Egypt)	−0.42	0.63	0.506	−1.67, 0.82	−1.35	0.85	0.114	−3.03, 0.33
Governmental hospital (ref. no)	0.06	0.55	0.914	−1.02, 1.13	0.57	0.74	0.444	−0.88, 2.02
Formal complaint (ref. lawsuit)	−1.08	0.59	0.065	−2.24, 0.07	**−1.81**	**0.79**	**0.022**	**−3.37, −0.26**
Threat or blackmail (ref. lawsuit)	−0.45	0.93	0.625	−2.28, 1.37	0.20	1.25	0.876	−2.27, 2.66
Procedure-related allegation (ref. no)	−0.79	0.59	0.176	−1.95, 0.36	−0.10	0.79	0.896	−1.66, 1.45
Diagnosis-related allegation (ref. no)	−0.92	0.61	0.133	−2.11, 0.28	−1.46	0.82	0.076	−3.07, 0.15
Medication-related allegation (ref. no)	−0.71	1.01	0.480	−2.69, 1.27	1.42	1.35	0.293	−1.23, 4.08
Unfavorable outcome (ref. favorable)	0.07	0.69	0.922	−1.29, 1.42	1.06	0.93	0.253	−0.76, 2.89
Still in progress (ref. favorable outcome)	−0.22	0.75	0.768	−1.69, 1.25	1.57	1.00	0.119	−0.41, 3.54

Correlate (reference)	With malpractice fear score (Table 7)				Without malpractice fear score			
	B	SE	p	95% CI	B	SE	p	95% CI
Personal support (ref. no)	0.12	0.63	0.847	−1.11, 1.35	0.16	0.85	0.850	−1.50, 1.82
Workplace support (ref. no)	0.24	0.58	0.678	−0.90, 1.39	−0.61	0.79	0.438	−2.16, 0.94
Psychological distress (K6 score)	0.09	0.06	0.161	−0.04, 0.21	**0.23**	**0.08**	**0.008**	**0.06, 0.39**
PTSS (IES-6 mean item score)	**0.93**	**0.46**	**0.045**	**0.02, 1.84**	**2.51**	**0.62**	**<0.001**	**1.29, 3.73**
Malpractice fear score	**3.32**	**0.15**	**<0.001**	**3.02, 3.63**	–	–	–	–
**Constant**	15.81				36.76			
**R^2; adjusted R^2**	0.566; 0.551				0.208; 0.181			
**F (df1, df2); p**	36.78 (20, 563); <0.001				7.78 (19, 564); <0.001			
**VIF range**	1.06–2.43				1.06–2.37			
**Maximum Cook's distance**	0.046				0.035			
**Breusch–Pagan p**	0.169				0.945			

B = unstandardized coefficient; CI = confidence interval; DMBS = Defensive Medicine Behavior Scale (12 items); IES-6 = Impact of Event Scale-6; K6 = Kessler Psychological Distress Scale-6; MBBCh = Bachelor of Medicine and Bachelor of Surgery; PTSS = post-traumatic stress symptoms; ref. = reference category; SE = standard error; VIF = variance inflation factor. The outcome of both models is the DMBS score; the second model omits the malpractice fear score. Bold: $p < 0.05$.

Table S12. Sensitivity analysis adjusted for specialty group (n = 586).

Outcome and correlate (reference)	Estimate	Main model (95% CI)	p	Specialty-adjusted (95% CI)	p
**Management-related allegation**					
Governmental hospital (ref. no)	AOR	0.67 (0.47–0.94)	0.020	**0.71 (0.50–1.00)**	**0.0498**
Other hospital # (ref. no)	AOR	0.50 (0.26–0.96)	0.039	**0.49 (0.25–0.96)**	**0.036**
Obstetrics and gynecology (ref. non-surgical)	AOR	–	–	0.72 (0.47–1.12)	0.148
Other surgical specialty (ref. non-surgical)	AOR	–	–	1.36 (0.89–2.09)	0.161
**Procedure-related allegation**					
Resident (ref. consultant)	AOR	0.27 (0.09–0.87)	0.029	**0.22 (0.07–0.73)**	**0.013**
Practice in another Arab country (ref. Egypt)	AOR	0.44 (0.29–0.67)	<0.001	**0.50 (0.32–0.76)**	**0.001**
Governmental hospital (ref. no)	AOR	1.50 (1.02–2.19)	0.038	1.41 (0.96–2.08)	0.081
University hospital (ref. no)	AOR	1.66 (1.01–2.73)	0.044	**1.80 (1.09–2.97)**	**0.022**
Other hospital # (ref. no)	AOR	2.33 (1.17–4.65)	0.017	**2.39 (1.19–4.78)**	**0.014**
Obstetrics and gynecology (ref. non-surgical)	AOR	–	–	**1.81 (1.14–2.90)**	**0.012**
Other surgical specialty (ref. non-surgical)	AOR	–	–	0.75 (0.47–1.19)	0.222
**Diagnosis-related allegation**					
Non-surgical specialty (ref. surgical)	AOR	1.81 (1.23–2.69)	0.003	replaced by specialty groups	–
Practice in another Arab country (ref. Egypt)	AOR	2.42 (1.63–3.59)	<0.001	**2.48 (1.66–3.71)**	**<0.001**
Other hospital # (ref. no)	AOR	0.39 (0.16–0.95)	0.037	**0.38 (0.16–0.93)**	**0.034**
Obstetrics and gynecology (ref. non-surgical)	AOR	–	–	**0.61 (0.38–0.98)**	**0.039**
Other surgical specialty (ref. non-surgical)	AOR	–	–	**0.51 (0.32–0.80)**	**0.004**
**Medication-related allegation**					
Female (ref. male)	AOR	2.31 (1.22–4.38)	0.010	**2.41 (1.18–4.90)**	**0.015**
Obstetrics and gynecology (ref. non-surgical)	AOR	–	–	0.55 (0.25–1.18)	0.122
Other surgical specialty (ref. non-surgical)	AOR	–	–	0.61 (0.28–1.37)	0.231
**Unfavorable versus favorable outcome (n = 484)**					
Specialist (ref. consultant)	AOR	1.80 (1.05–3.09)	0.032	1.71 (0.99–2.94)	0.052
Formal complaint (ref. lawsuit)	AOR	0.30 (0.18–0.49)	<0.001	**0.29 (0.17–0.48)**	**<0.001**

Outcome and correlate (reference)	Estimate	Main model (95% CI)	p	Specialty-adjusted (95% CI)	p
Threat or blackmail (ref. lawsuit)	AOR	0.45 (0.23–0.90)	0.023	**0.42 (0.21–0.84)**	**0.014**
Obstetrics and gynecology (ref. non-surgical)	AOR	–	–	**2.07 (1.16–3.67)**	**0.013**
Other surgical specialty (ref. non-surgical)	AOR	–	–	1.19 (0.66–2.16)	0.569
**Psychological distress (K6)**					
Age ≥ 43 years (ref. < 43 years)	B	−1.68 (−3.21, −0.16)	0.031	**−1.68 (−3.21, −0.14)**	**0.033**
Female (ref. male)	B	1.58 (0.44, 2.73)	0.007	**1.59 (0.34, 2.85)**	**0.013**
Private hospital or clinic (ref. no)	B	−1.57 (−2.58, −0.56)	0.002	**−1.56 (−2.58, −0.54)**	**0.003**
Diagnosis-related allegation (ref. no)	B	1.41 (0.29, 2.52)	0.013	**1.42 (0.29, 2.55)**	**0.014**
Personal support (ref. no)	B	1.27 (0.09, 2.46)	0.035	**1.26 (0.06, 2.45)**	**0.039**
Professional support (ref. no)	B	2.36 (1.25, 3.47)	<0.001	**2.37 (1.24, 3.49)**	**<0.001**
Obstetrics and gynecology (ref. non-surgical)	B	–	–	0.15 (−1.25, 1.54)	0.837
Other surgical specialty (ref. non-surgical)	B	–	–	0.19 (−1.12, 1.49)	0.778
**PTSS (IES-6)**					
Female (ref. male)	B	0.20 (0.04, 0.37)	0.015	**0.18 (0.00, 0.36)**	**0.047**
Specialist (ref. consultant)	B	0.23 (0.04, 0.43)	0.018	**0.24 (0.04, 0.43)**	**0.018**
Management-related allegation (ref. no)	B	0.17 (0.02, 0.31)	0.023	**0.17 (0.03, 0.32)**	**0.020**
Diagnosis-related allegation (ref. no)	B	0.25 (0.10, 0.40)	0.001	**0.25 (0.10, 0.41)**	**0.001**
Professional support (ref. no)	B	0.30 (0.15, 0.45)	<0.001	**0.31 (0.16, 0.46)**	**<0.001**
Obstetrics and gynecology (ref. non-surgical)	B	–	–	0.07 (−0.12, 0.26)	0.458
Other surgical specialty (ref. non-surgical)	B	–	–	0.03 (−0.15, 0.21)	0.733
**Somatic symptom score**					
Female (ref. male)	B	1.15 (0.63, 1.66)	<0.001	**0.86 (0.30, 1.42)**	**0.003**
Not married (ref. married)	B	−1.18 (−1.89, −0.46)	0.001	**−1.15 (−1.86, −0.44)**	**0.002**
Practice in another Arab country (ref. Egypt)	B	−0.85 (−1.36, −0.34)	0.001	**−0.77 (−1.29, −0.26)**	**0.003**
More than one dispute (ref. one)	B	0.67 (0.21, 1.13)	0.004	**0.68 (0.22, 1.14)**	**0.004**
Formal complaint (ref. lawsuit)	B	−0.64 (−1.12, −0.17)	0.008	**−0.66 (−1.14, −0.19)**	**0.006**
Workplace support (ref. no)	B	−0.53 (−1.02, −0.04)	0.033	−0.48 (−0.97, 0.00)	0.052
Obstetrics and gynecology (ref. non-surgical)	B	–	–	**0.61 (0.02, 1.21)**	**0.043**
Other surgical specialty (ref. non-surgical)	B	–	–	−0.17 (−0.72, 0.38)	0.551
**Defensive medicine (DMBS)**					
MBBCh only (ref. MD/PhD/fellowship/board)	B	4.24 (0.14, 8.33)	0.043	**4.47 (0.34, 8.60)**	**0.034**
PTSS (IES-6 mean item score)	B	0.93 (0.02, 1.84)	0.045	**0.92 (0.01, 1.83)**	**0.049**
Malpractice fear score	B	3.32 (3.02, 3.63)	<0.001	**3.32 (3.01, 3.62)**	**<0.001**
Obstetrics and gynecology (ref. non-surgical)	B	–	–	0.40 (−1.01, 1.81)	0.577
Other surgical specialty (ref. non-surgical)	B	–	–	−0.19 (−1.52, 1.15)	0.783
**Malpractice fear score**					
More than one dispute (ref. one)	B	0.42 (0.12, 0.73)	0.006	**0.43 (0.13, 0.73)**	**0.005**
Medication-related allegation (ref. no)	B	0.59 (0.05, 1.12)	0.032	**0.60 (0.06, 1.13)**	**0.029**
Still in progress (ref. favorable outcome)	B	0.60 (0.20, 0.99)	0.003	**0.61 (0.22, 1.01)**	**0.002**
Psychological distress (K6 score)	B	0.04 (0.01, 0.08)	0.013	**0.04 (0.01, 0.08)**	**0.010**
PTSS (IES-6 mean item score)	B	0.46 (0.22, 0.70)	<0.001	**0.45 (0.21, 0.69)**	**<0.001**
Obstetrics and gynecology (ref. non-surgical)	B	–	–	0.30 (−0.08, 0.69)	0.124
Other surgical specialty (ref. non-surgical)	B	–	–	−0.02 (−0.39, 0.34)	0.903

AOR = adjusted odds ratio; B = unstandardized coefficient; CI = confidence interval; DMBS = Defensive Medicine Behavior Scale; IES-6 = Impact of Event Scale-6; K6 = Kessler Psychological Distress Scale-6; PTSS = post-traumatic stress symptoms. Each main model (Tables 3, 4, 6 and 7) was re-fitted with specialty in three groups (obstetrics and gynecology, other surgical specialties, and non-surgical specialties as

reference), replacing the two-group specialty variable where it was present; the two participants without a specified specialty were excluded. The table shows every correlate with $p < 0.05$ in the main model and the specialty-group estimates. Bold: $p < 0.05$; a p value between 0.0495 and 0.05 is shown with four decimals.
